# Identification of a novel chalcone derivative that inhibits Notch signaling in T-cell acute lymphoblastic leukemia

**DOI:** 10.1038/s41598-017-02316-9

**Published:** 2017-05-19

**Authors:** Mattia Mori, Luca Tottone, Deborah Quaglio, Nadezda Zhdanovskaya, Cinzia Ingallina, Marisa Fusto, Francesca Ghirga, Giovanna Peruzzi, Maria Elisa Crestoni, Fabrizio Simeoni, Francesca Giulimondi, Claudio Talora, Bruno Botta, Isabella Screpanti, Rocco Palermo

**Affiliations:** 1 Center for Life Nano Science@Sapienza, Istituto Italiano di Tecnologia, Rome, 00161 Italy; 2 Department of Molecular Medicine, Sapienza University of Rome, Rome, 00161 Italy; 3 Department of Chemistry and Technology of Drugs, Sapienza University of Rome, Rome, 00185 Italy; 4 Istituto Pasteur Fondazione Cenci Bolognetti, Sapienza University of Rome, Rome, 00161 Italy; 5 Cancer Research UK Manchester Institute, The University of Manchester, Manchester, M20 4BX UK

## Abstract

Notch signaling is considered a rational target in the therapy of several cancers, particularly those harbouring Notch gain of function mutations, including T-cell acute lymphoblastic leukemia (T-ALL). Although currently available Notch-blocking agents are showing anti-tumor activity in preclinical studies, they are not effective in all the patients and often cause severe side-effects, limiting their widespread therapeutic use. Here, by functional and biological analysis of the most representative molecules of an *in house* library of natural products, we have designed and synthetized the chalcone-derivative **8** possessing Notch inhibitory activity at low micro molar concentration in T-ALL cell lines. Structure-activity relationships were afforded for the chalcone scaffold. Short term treatments with compound **8** resulted in a dose-dependent decrease of Notch signaling activity, halted cell cycle progression and induced apoptosis, thus affecting leukemia cell growth. Taken together, our data indicate that **8** is a novel Notch inhibitor, candidate for further investigation and development as an additional therapeutic option against Notch-dependent cancers.

## Introduction

The Notch signaling pathway is an inter-cellular communication system driving many biological processes starting from stem cells self-renewal up to cell differentiation, proliferation and survival in different tissues and in a wide spectrum of organisms^1^. The mammalian Notch family includes four highly evolutionarily conserved trans-membrane receptors (Notch1–4) and five canonical ligands (Jagged-1, and -2, Delta-like-1, -3 and -4).

Notch proteins are synthetized as immature precursors in Endoplasmic Reticulum. Following the proteolytic cleavage by Furin-like convertase (S1 cleavage) in the trans-Golgi, mature Notch receptors accumulate on cell surface as heterodimers composed of the Notch extracellular domain (NECD), the transmembrane domain (NTM) and the intracellular domain (NICD), held together by non-covalent interactions. Notch signaling-induced trans-activation is triggered by the contact between a membrane-associated ligand on the signal-sending cell and a Notch trans-membrane receptor on the signal-receiving cell. The interaction with the ligand predisposes the Notch receptor to the cleavage by ADAM metalloproteases (S2 cleavage), that allows the subsequent cleavage by gamma secretase (GS) complex (S3 cleavage). S3 cleavage leads to the release of active NICD from the membrane, which translocates to the nucleus and regulates the transcription of specific target genes^2^.

Deregulated Notch signaling due to either gene mutation or amplification, or to post-translational modifications, contributes to development and progression of different solid and hematological cancers, including T-cell acute lymphoblastic leukemia (T-ALL)^3^, by directly driving the expression of several oncogenes and cell cycle-related factors^4–7^, and indirectly by cross-talking with other critical oncogenic signaling pathways^8–12^.

T-ALL arises in the thymus from the malignant transformation of T-cell progenitors and is one of the most aggressive blood cancers, which accounts for approximately 15% of paediatric and 25% of adult acute lymphoblastic leukemia. Although T-ALL prognosis has gradually improved, thanks to the available chemotherapeutic protocols, to date the fate of patients with primary therapy-resistant or relapsed leukemia remains unfavourable^13, 14^.

Several Notch-blocking agents have been developed up to preclinical research and a number of them have been moved to clinical trials for the therapy of Notch-driven tumors, including T-ALL^15^. In this respect, it is worth noting that the most promising pharmacologic approach to block Notch signaling relies in the suppression of the S3 cleavage operated by GS, which leads to the generation of the NICD. Unfortunately, as revealed by clinical trials, the potential clinical applications of GS inhibitors (GSIs) is limited by primary resistance and/or by severe side effects, especially those occurring within the gastrointestinal tract^16^.

In the last decade, naturally occurring chemotypes re-emerged as lead candidates for anticancer therapy^17–20^ and, a number of natural products affecting Notch gene expression have been suggested as potential anti-cancer Notch inhibitors^21–23^.

In the present study, an *in house* library composed of about one thousand natural products and their chemical derivatives^24–27^ was clustered based on fingerprints and substructure search through a cheminformatics approach^28^. The representative compounds of the eight most populated clusters have been screened by functional, biological and biochemical investigations, for their strength in impairing Notch signaling activity and cell growth in Notch-dependent human T-ALL cell lines. The 2′,3,4,4′-tetrahydroxychalcone (molecule **C**, butein) emerged as valuable hit compound, thus emphasizing the relevance of the chalcone scaffold in Notch inhibition. However, the molecule contains a catechol moiety, which is susceptible for oxidation both *in vitro* and *in vivo*, providing possible off-target effects^29^. Therefore, cycles of design-synthesis-bioassay and testing *in vitro* were established to optimize the potency of initial chalcone hit, and to eliminate or mask the undesirable chemical feature. Structure-activity relationships (SAR) were afforded, and a novel potent Notch inhibitor, namely **8**, was identified and characterized. Compound **8** short term and low dose treatments inhibited the endogenous Notch signaling activity and suppressed cell growth of several human T-ALL cell lines by promoting apoptosis and G1 cell cycle arrest.

Interestingly, compound **8** inhibits Notch signaling without interfering with S2 and S3 proteolytic cleavages, depending on ADAM and GS, respectively. Overall, our findings suggest molecule **8** as a novel Notch inhibitor candidate for further investigation and development.

## Results

### Butein is a naturally occurring Notch signaling inhibitor

An *in house* library of about one thousand natural products and their derivatives was used as source of potential modulators of the Notch signaling. Notably, natural products have long been used as medicines and remedies for human disease, and in the modern era are considered as a relevant source of lead compounds for drug discovery^19, 20, 25, 30^. Accordingly, our *in house* library is a valid source of chemotypes for the modulation of biomolecular targets, and it has been successfully screened *in silico* and *in vitro* for the identification of hit and lead compounds in previous early-stage drug discovery projects^24–27^.

Here, to concentrate experimental efforts on a relatively low number of molecules, and to explore as much as possible the chemical and scaffold space of the library, we envisaged to test *in vitro* a representative subset of molecules. To this end, a diversity-oriented random selection (DORS) of compounds was performed by means of a clustering algorithm, which is based on a combination of fingerprints and common substructure (adapted from that developed by Stahl and Mauser)^28^. The representative molecules of the eight most abundant clusters were selected for this work (Table 1), based on physical availability at the time of the experiments, hit-like features^31^, and chemical diversity criteria.

**Table 1:** Structure and inhibitory activity of natural compounds **A**–**H**.

Compound	Chemical structure	IC_50_ ± S.D. (μM)
A		>50
B		13.89 ± 2.70
C		7.79 ± 1.73
D		>50
E		>50
F		>50
G		>50
H		22.64 ± 1.89

IC_50_ values were determined towards DND41 cells at 36 hrs incubation times. Data represent mean of values for triplicates ± standard deviations (S.D.).

As a useful experimental model to screen putative Notch inhibitors we utilized DND41 human T-ALL cells which harbour constitutively activated Notch1 signaling due to activating Notch1 gene mutations^3^ and express high levels of Notch3^6^. In addition, the growth of DND41 cells is sensitive to the inhibition of Notch signaling by the treatment with GSI^32^.

**Figure 1:**
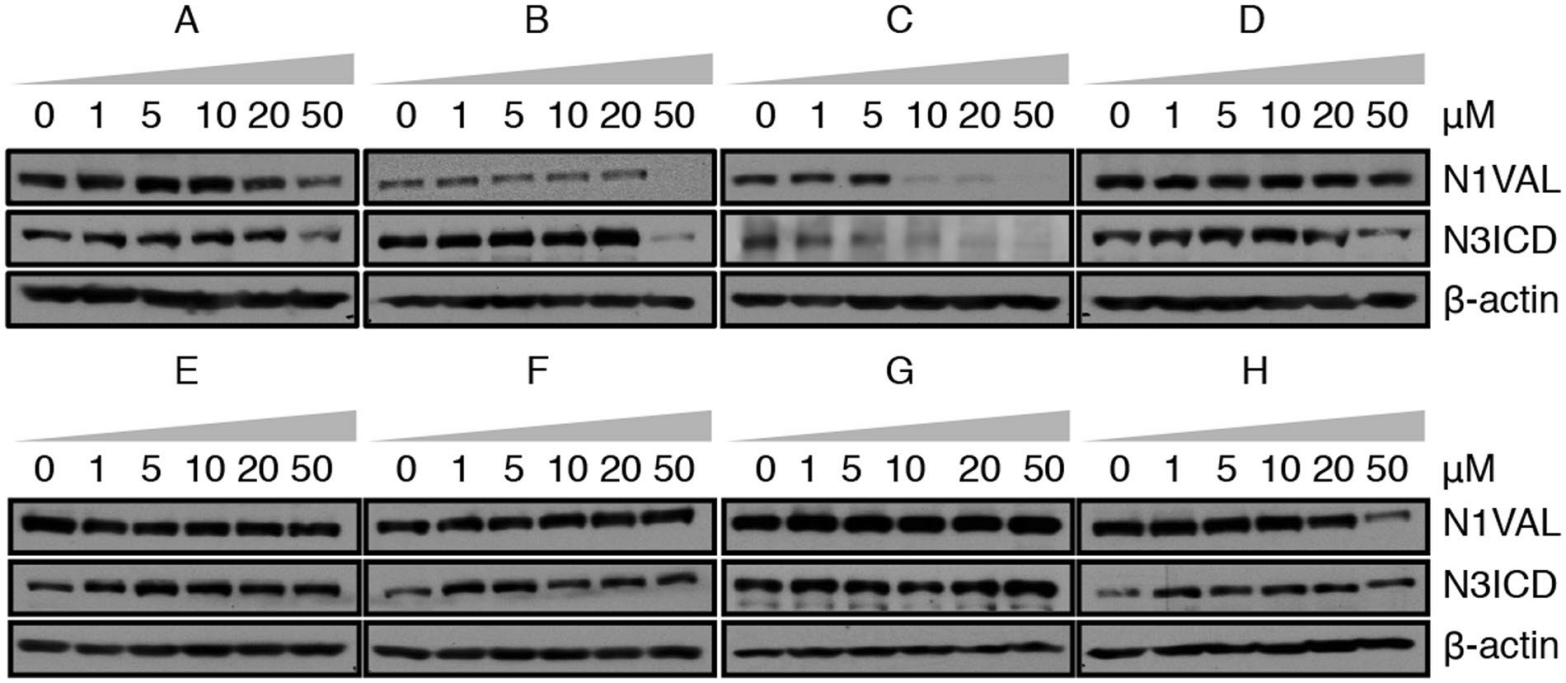
Chalcone **C** inhibits endogenous Notch signaling activation in DND41 T-ALL cells. Activated Notch1 receptor domain (N1VAL) and Notch3 intracellular domain (N3ICD) expressions in DND41 T-ALL cell lines in response to 36 hrs of different doses exposure to eight molecules (named **A**–**H**) representative of the *in house* library consisting of one thousand natural compounds. β-actin is used as loading control. Uncropped Western blots related to this figure are displayed in Fig. S13.

To identify novel Notch modulating agents, we evaluated the effects on the endogenous Notch1 activation in DND41 human cells after 36 hrs of exposure to increasing doses of selected molecules (0, 1, 5, 10, 20, 50 μM). Treatments with compounds **D**, **E**, **F** and **G** did not influence the expression of the activated domain of Notch1, as revealed by western blot by using the antibody against the Valine 1744 (N1VAL), whereas the exposure to compounds **A**, **B** and **H** resulted in its decrease only at high doses (between 20 μM and 50 μM). On the other hand, compound **C** showed the highest Notch inhibiting activity as revealed by decreased immunoreactivity to N1VAL at low micro molar concentration (between 5 μM and 10 μM) (Fig. 1). Effectiveness of Notch signaling modulation was further confirmed by the decreased levels of expression of the intracellular domain of Notch3 receptor (N3ICD).

To investigate the biological implications of the above compounds, we then tested their ability to exert anti-proliferative effects. Thus, we evaluated the growth sensitivity of DND41 T cells to these compounds by determining the half-maximal growth inhibitory concentration (IC_50_) by MTS cell assay. In keeping with its highest Notch blocking activity, the compound **C** was the most powerful molecule in inhibiting cell growth with IC_50_ value of 7.79 ± 1.73 μM (Table 1 and Fig. S1).

By contrast, compounds **B** and **H** showed a weaker growth inhibitory activity with IC_50_ values of 13.89 ± 2.70 μM and 22.64 ± 1.89 μM, respectively, while compounds **A**, **D**, **E**, **F** and **G** did not influence considerably DND41 cell growth up to 50 μM (Table 1 and Fig. S1). Cross-comparative assessment of the above data led us to select, among the screened compounds, the chalcone **C** as the most powerful Notch inhibitor.

### Design and synthesis of chemical derivatives of butein

**Figure 2:**
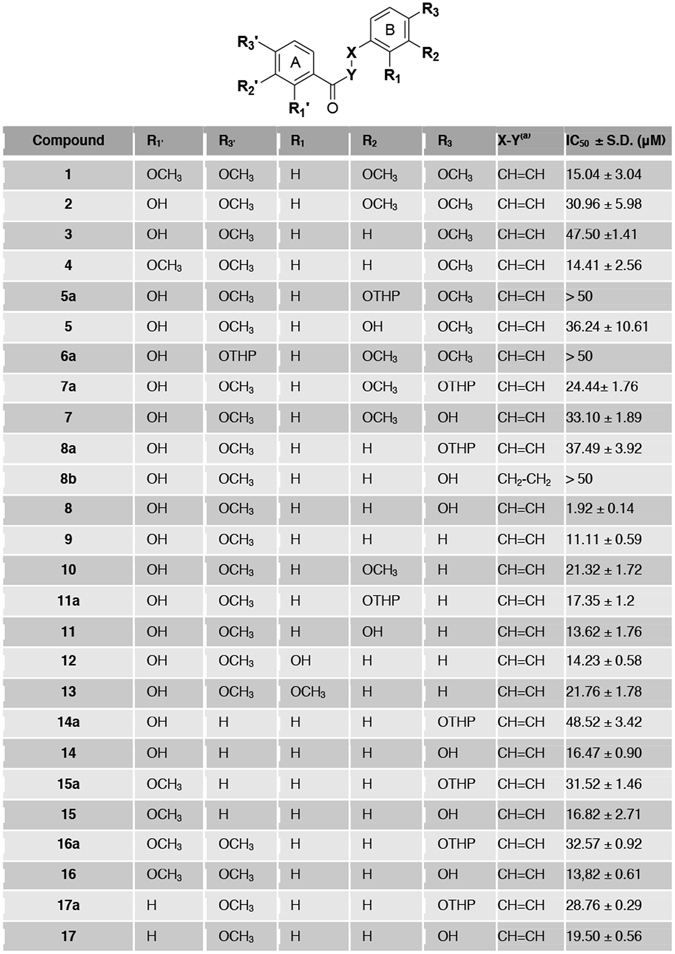
Structure and inhibitory activity of chalcone derivatives **1**–**17**. IC_50_ values were determined towards DND41 cells at 36 hrs incubation times. Data represent mean of values for triplicates ± standard deviations (S.D.). ^a^All double bonds are in *E* configuration.

The chalcone scaffold emerged as a promising tool to inhibit Notch signaling, and was therefore selected for further investigations. With the aim of increasing the potency of the hit compound **C**, to remove the undesired catechol moiety^29^, and to afford SAR that may facilitate further development as well as the understanding of the molecular determinants for Notch inhibition, compound **C** derivatives **1**, **2**, **3**, **4**, **5**, **5a**, **6a**, **7**, **7a**, **8**, **8a** and **9** were designed and synthesized (Fig. 2).

**Figure 3:**
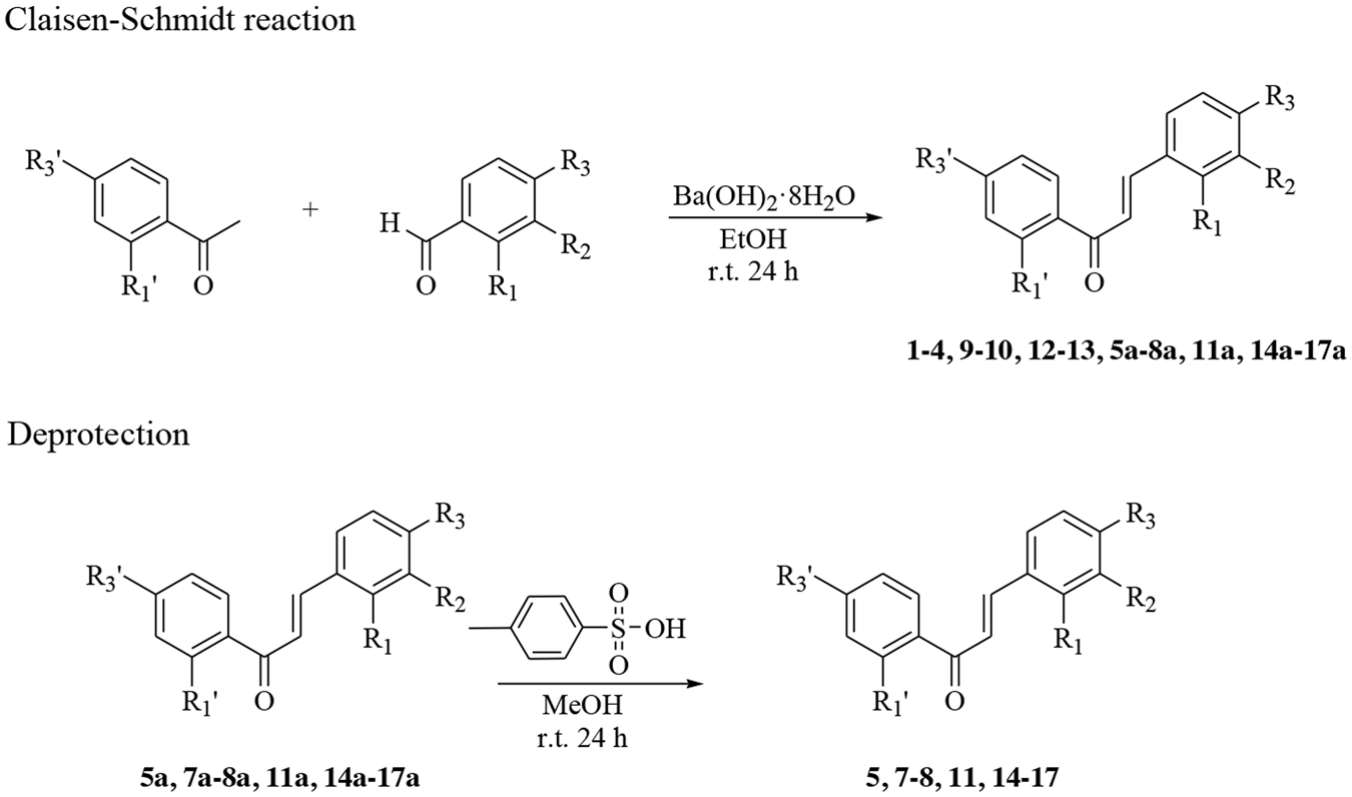
General synthesis of chalcone derivatives **1**–**17**. The synthetic pathway is based on the Claisen-Schmidt condensation reaction. Selected compounds **5a**, **7a–8a**, **11a**, **14a–17a** were deprotected to give the corresponding chalcones.

The general strategy employed to synthesize chalcones in excellent yield was based on the Claisen-Schmidt condensation as reported previously^33^ and illustrated in Fig. 3.

### Functional and biological evaluation of the Notch signaling inhibitory activity in T-ALL of the newly synthesized chalcones

**Figure 4:**
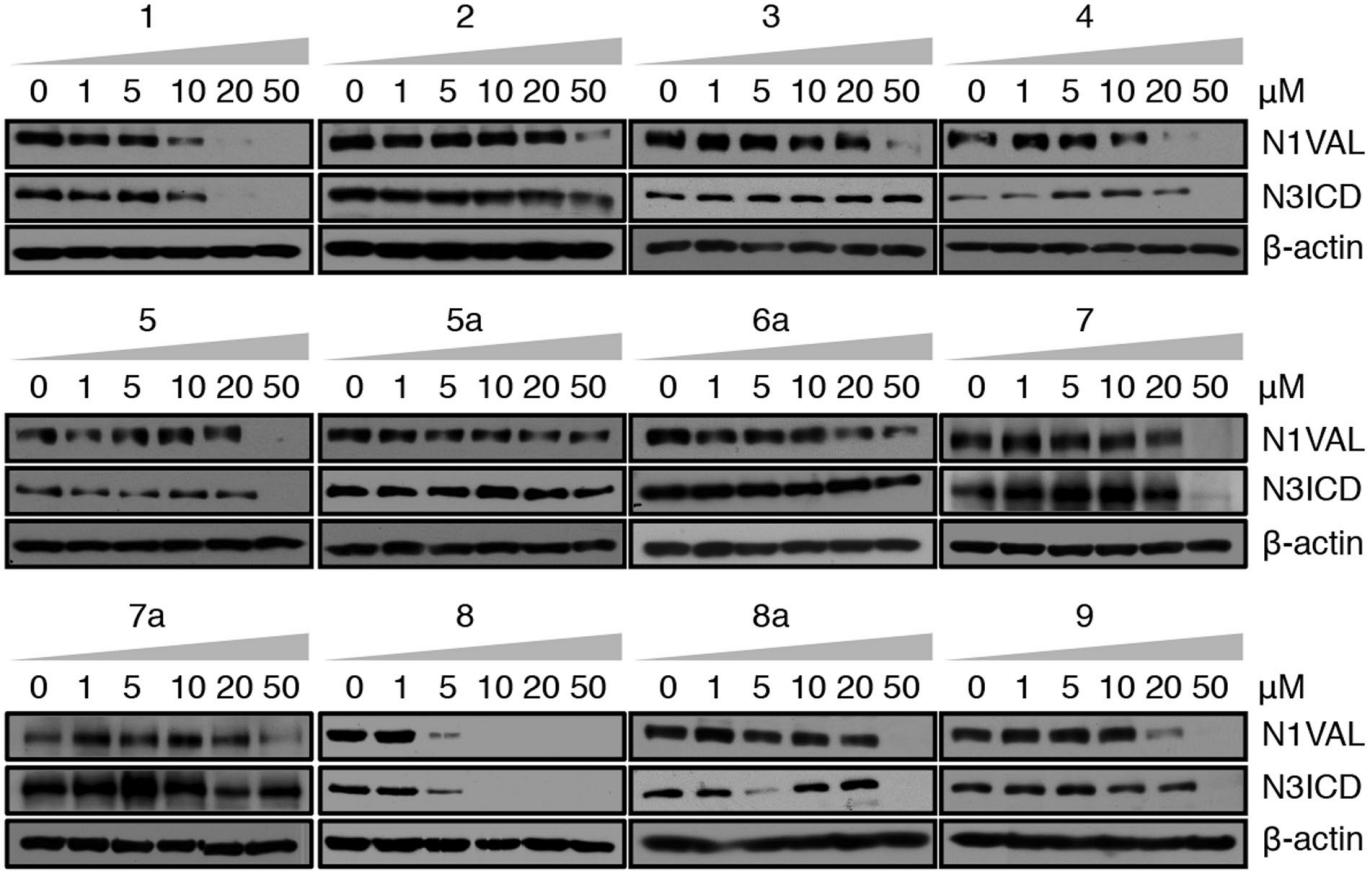
Compound **8** is a potent Notch signaling inhibitor. Endogenous N1VAL and N3ICD protein expression levels in DND41 T-ALL cells in response to 36 hrs of different doses of exposure to the twelve **C**-derivative molecules (named **1**, **2**, **3**, **4**, **5**, **5a**, **6a**, **7**, **7a**, **8**, **8a** and **9**). β-actin is used as a loading control. Uncropped Western blots related to this figure are displayed in Fig. S14.

To probe the effectiveness of the above twelve derivatives of chalcone **C**, we investigated, in a dose-dependent manner at concentrations of 0, 1, 5, 10, 20, 50 μM, their potency as Notch signaling inhibitors in DND41 cells. Among them, compound **8** revealed improved potency and specificity of action compared to chalcone **C**, as it decreased N1VAL and N3ICD protein expression levels at the lowest micro molar doses (between 1 μM and 5 μM). In contrast, all the remaining synthetic derivatives modulated N1VAL and N3ICD expression levels at higher doses than the hit chalcone **C** (Fig. 4). Accordingly, dose–response cell proliferation assay proved that compound **8** was the most effective molecule in impairing DND41 T-cell growth, with an IC_50_ value of 1.92 μM ± 0.14 μM. All the other chemical derivatives were 10–20 times weaker than compound **8**, as they showed IC_50_ values higher than 11 μM (Fig. 2). Substantial inhibition of cell growth was also reflected in molecule **8**-treated cells growth curve (Fig. S2).

Given the high power of action at low micro molar concentration of compound **8**, the subsequent functional and biological comparisons between compound **8** and fourteen newly-synthesized compounds were performed including two lower doses to the above showed dose response assays (0.5 μM and 2.5 μM).

Among the newly synthesized derivatives of compound **8**, molecules **16** and **17** showed the strongest Notch signaling inhibitory activity in terms of potency in impairing endogenous Notch activity in DND41 cells, in a range of concentrations comparable to those shown by compound **8** (2.5 μM) (Fig. S3a). The treatments with the remaining derivatives of **8** resulted in weaker inhibition of Notch signaling activity, as all of them decreased N1VAL and N3ICD expressions at doses higher than 5 μM (Fig. S3a). Moreover, the subsequent close comparison of the Notch inhibitory potency at 2.5 μM between compounds **8**, **16** and **17** confirmed, among them, **8** as the strongest Notch signaling inhibitor (Fig. S4). In addition, compound **8** confirmed the strongest biological activity as reflected by the cell growth curves and the relative IC_50_ values, in response to increasing doses of all the synthetic derivatives (Figs S3b and 2). Indeed, all derivatives of **8**, including **16** and **17**, showed IC_50_ values higher than 13 μM (Fig. 2). Interestingly, among tested molecules, **8b** provided the poorer effects on both Notch signaling inhibition and cell growth as showed by the western blots against N1VAL and N3ICD, and by the growth curve and IC_50_ values (Figs S3 and 2).

### SAR of chalcone derivatives as Notch inhibitors

**Figure 5:**
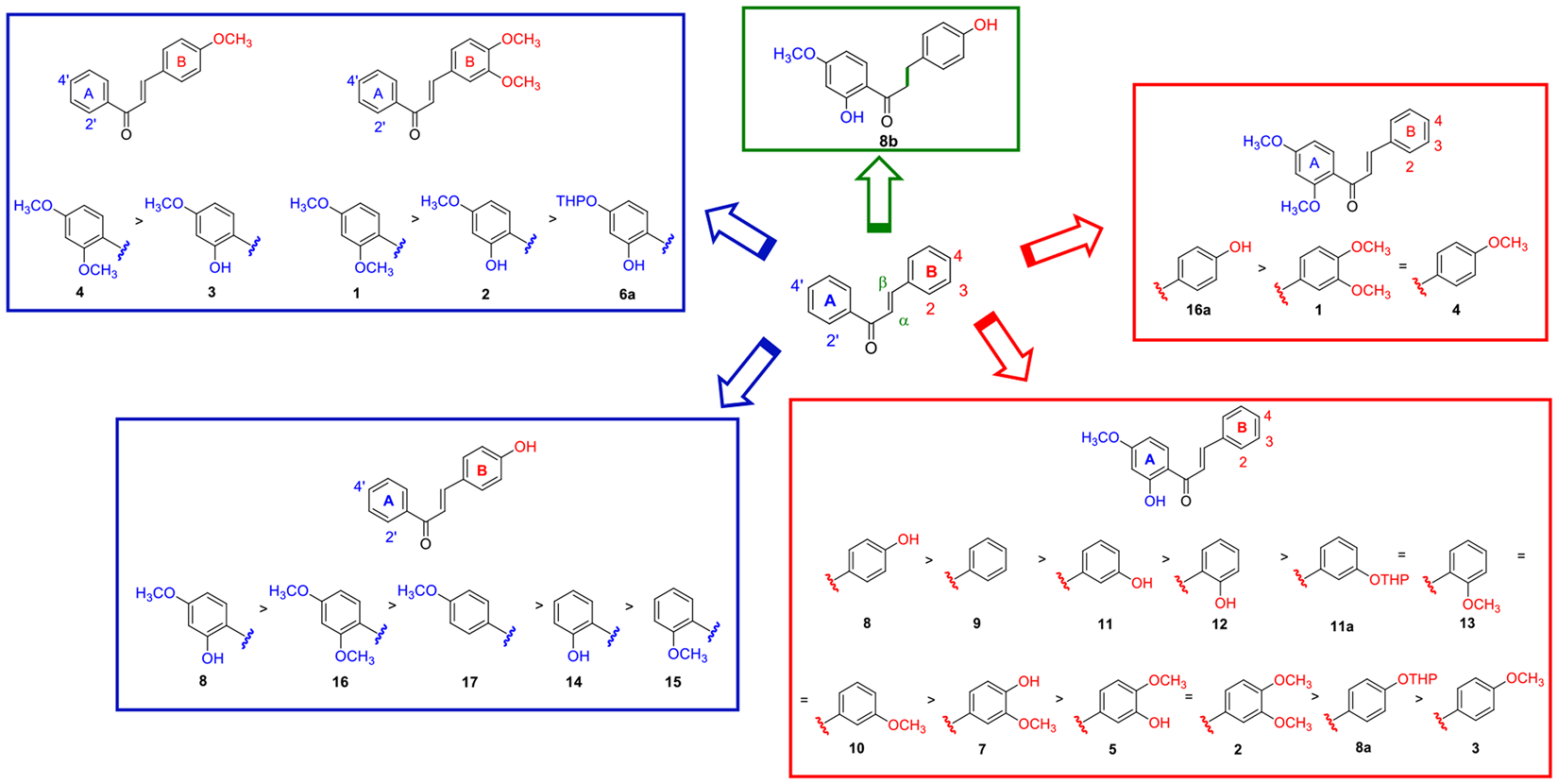
SAR of the chalcone derivatives as Notch inhibitors in T-ALL. Three structural levels were identified by analyzing functional and biological data: ring A, ring B and α, β-unsaturated carbonyl system of the chalcone scaffold.

Functional and biological analysis of the naturally occurring chalcone **C** and its newly synthesized twenty-six chemical derivatives highlighted the molecule **8** as the most active Notch inhibitor in T-ALL. SAR were elaborated at three structural levels: ring A, ring B and the α,β-unsaturated carbonyl system of the chalcone scaffold, and were graphically sketched in Fig. 5.

Concerning ring A, methylation of the hydroxyl group at position 2′ improved the inhibitory effect on cell proliferation only when ring B is not substituted with hydroxyl group at position 4. In this condition, the presence of methoxyl group at position 4′ provides higher activity than the hindered tetrahydropyranyl protecting group. Therefore, since chalcone **8** is the most potent Notch inhibitor of the series, the 2′ and 4-hydroxy groups seem to act synergistically. Based on this evidence, the mono substitution of ring A in different positions (**14**, **15** and **17**) originates weaker chalcones compared to the 2′,4′-substitutions (**8** and **16**). Finally, mono substituted derivatives showed similar inhibitory effect on cell viability, even though the 4′-methoxy group (**17**) drastically reduced the N1VAL and N3ICD expression in a comparable extent to **8**, and in contrast to what observed for 2′-substituted chalcones (**14** and **15**) (Figs 5 and S3).

Notably, the potency of chalcone derivatives seems to be significantly dependent from the pattern of substitutions to ring B. In fact, comparing chalcones bearing the same ring A, the hydroxyl group improved the activity of the molecules particularly when it is inserted at position 4 of ring B, which proved to be critical for reducing both cell proliferation and Notch expression (**8**) (Figs 5 and S3).

Finally, the reduction of the double bond in the α, β-unsaturated carbonyl system (**8b**) completely abrogates biological effects, thus suggesting that the restricted flexibility of the spacer in chalcones is required to keep the pharmacophoric phenyl rings A and B at the suitable distance to provide potent Notch inhibition.

### Compound 8-induced Notch signaling inhibition suppresses several leukemia cell lines growth by promoting apoptosis and G1 cell cycle arrest

To preliminarily investigate if compound **8** inhibits Notch signaling by suppressing endogenous GS or ADAM metalloprotease enzymatic activities, we compared the effects of compound **8** with the GSI DAPT or with the ADAM10 specific inhibitor GI254023X (GI) on the transcriptional activity of a membrane-tethered Notch1 intracellular domain, including the aminoacidic sequence of the region surrounding the ADAM cleavage site (N1ICD-ΔE)^34^. HEK cells were transiently co-transfected with a Notch responsive 12X CSL-Luciferase reporter and the construct encoding N1ICD-ΔE. Transfected cells were then exposed for 36 hrs to increasing doses of compound **8**, DAPT or GI. Compound **8** treatments did not affect the transcriptional activity. Conversely, as expected, both DAPT and GI exposure decreased the promoter luciferase activity in N1ICD-ΔE co-transfected cells (Fig. S5). Overall, our results revealed that compound **8** does not act by inhibiting GS- or ADAM-dependent Notch cleavages.

We next explored, the possibility that compound **8** affects Notch receptor transport to the membrane through the Golgi. To this aim, we investigated the surface expression of Notch1 and Notch3 receptors upon treatments with compound **8** or DAPT. DND41 cells treated with compound **8** show slight increased Notch1 surface expression, when compared to the vehicle-treated cells, while DAPT treatments resulted in greater surface Notch1 receptor accumulation, due to the S3 blockade (Fig. S6). On the other hand, both treatments compound **8** and DAPT, decreased Notch3 surface expression in DND41 cells (Fig. S6). Notably, decreased Notch3 protein surface expression in DND41 cells treated with compound **8** is paralleled by decreased Notch3 gene expression upon compound **8** treatments in DND41 cell lines, in which Notch3 is a Notch1 target gene^35^ (Fig. S7).

**Figure 6:**
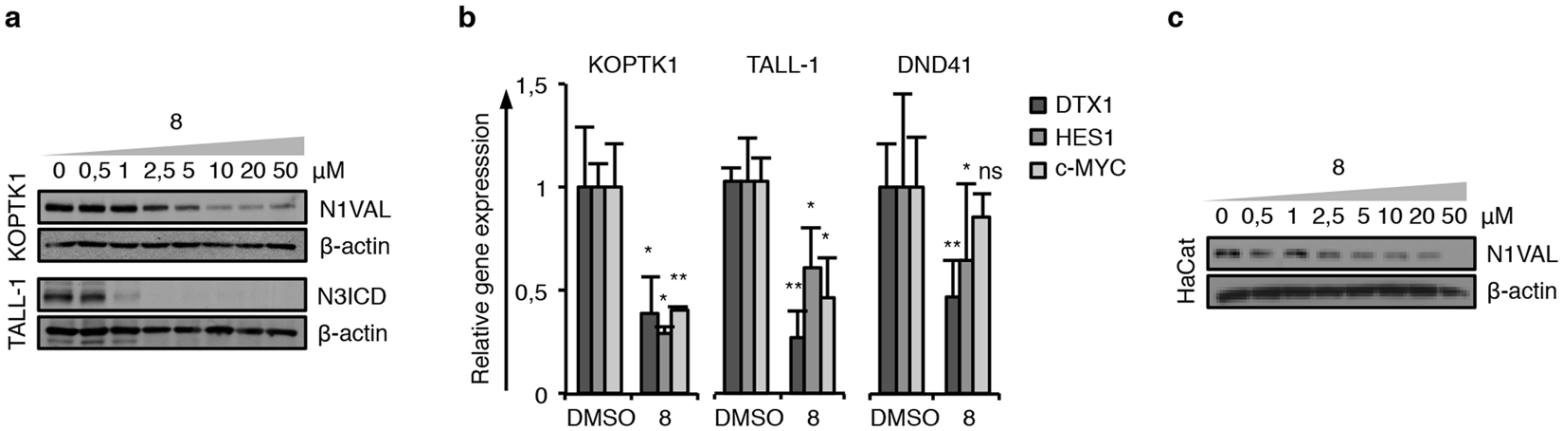
Compound **8** counteracts Notch signaling in T-ALL cell contexts. (**a**) N1VAL, N3ICD and β-actin protein expression levels in response to 36 hrs of different doses of compound **8** exposures in KOPTK1 and TALL-1. (**b**) Relative DELTEX1 (DTX1), HES1 and c-MYC gene expression levels in response to 36 hrs of treatment with molecule **8** in KOPTK1, TALL-1 and DND41 cells. In detail, KOPTK1 and DND41 cell were treated with 2.5 μM, while TALL-1 cell with 1 μM of compound **8**. Data represent mean values ± S.D. normalized to GAPDH of three independent experiments. *P < 0.05; **P < 0.01; ns not significant. (**c**) N1VAL and β-actin protein expression levels in response to 36 hrs of dose dependent manner of compound **8** exposures in HaCat cells. Uncropped Western blots related to this figure are displayed in Fig. S15.

Overall, the data above suggested compound **8** as the most promising Notch-blocking agent among the molecules screened, and in order to exclude cell-line-specific effects, we extended the analyses to additional T-ALL cell lines. Confirming its effectiveness on Notch signaling inhibition, 36 hrs of treatment with molecule **8**, as well as with DAPT, utilized as a positive control, decreased in a dose dependent manner the level of expression of N1VAL in DND41 and KOPTK1 and of N3ICD in TALL-1 cell lines characterized by constitutively active Notch1 or Notch3 signaling^36, 37^, respectively (Figs 4, 6a and S8).

Consistently, compound **8**-dependent Notch inhibition in DND41, KOPTK1 and TALL-1 cells correlated with the significant endogenous mRNA reduction of the known Notch target genes HES1^38^, DELTEX1^39^, c-MYC^4^ and NOTCH3^4^ (Figs 6b and S7) and the growth inhibition at low doses (KOPTK1: IC_50_ = 0.91 ± 0.06 μM and TALL-1: IC_50_ = 0.29 ± 0.02 μM).

Notably, 36 hrs of DAPT exposures up to 50 μM did not affect the proliferation of T-ALL cell lines (DND41: IC_50_ = >50 μM; KOPTK1: IC_50_ = >50 μM; TALL-1: IC_50_ = >50 μM). Interestingly, compound **8** and DAPT, did not affect cell proliferation in immortalized non-tumorigenic human keratinocyte HaCat cells in which Notch signaling acts as onco-suppressor^40^. Indeed, although compound **8** and DAPT treatments in HaCat cells decreased N1VAL protein expression in a dose dependent manner (Figs 6c and S8), none of them was able to exert substantial cell growth inhibitory effects (**8**: IC_50_ = 48.61 ± 8.06 μM; DAPT: IC_50_ = >50 μM). Consistently, compound **8** did not affect cell cycle progression and apoptosis rate in the same cells (Fig. S9).

**Figure 7:**
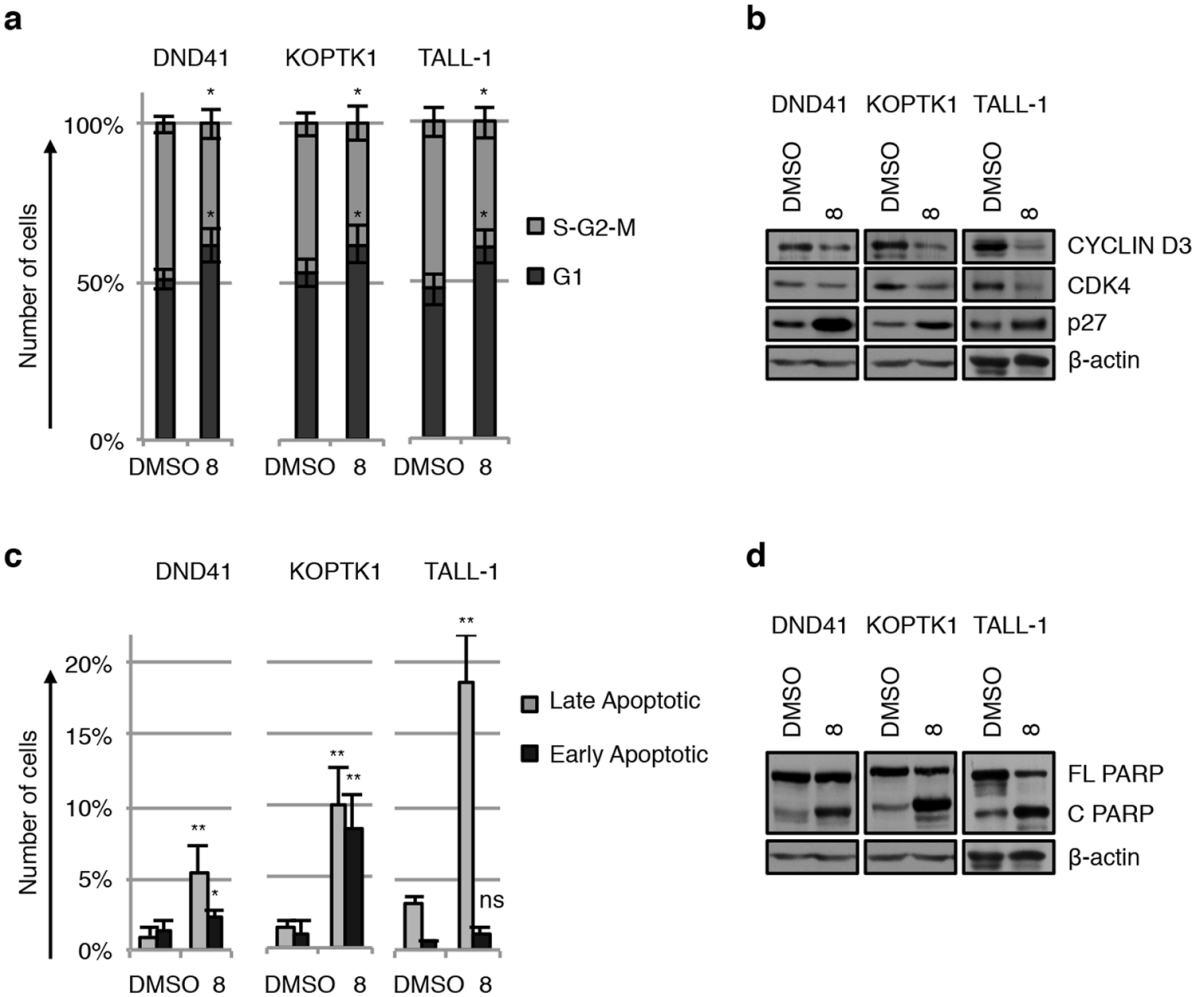
Compound **8**-treatment halts cell cycle in G_1_ phase and induces apoptosis in human T-ALL cells. T-ALL cell lines were treated for 36 hrs with compound **8** or with vehicle alone (DMSO) as indicated: DND41 and KOPTK1 with 2.5 μM of **8** and TALL-1 with 1 μM of **8**. Cell cycle progression was investigated by FACS analysis of DNA content after 7AAD staining. (**a**) Histograms show the mean of cell percentage ± S.D. in G1 versus S-G2-M phases of cell cycle of four independent experiments. *P < 0.05. (**b**) Protein expression levels of Cyclin D3, CDK4 and p27 in DND41, KOPTK1 and TALL-1 cells treated with compound **8** or with vehicle alone (DMSO). Compound **8**-induced apoptosis rate and cell death were investigated by FACS analysis after Annexin V and 7AAD staining in DND41, KOPTK1 and TALL-1 cells. (**c**) Histograms show the mean of cell percentage ± S.D. of early and late apoptotic cells of at least four independent experiments. *P < 0.05; **P < 0.01; ns not significant. (**d**) Compound **8**-induced effects on PARP activation in DND41, KOPTK1 and TALL-1 cells as shown by the protein expression levels of the precursor (FL PARP) and the cleaved PARP (C PARP). β-actin is used as loading control. Uncropped Western blots related to this figure are displayed in Fig. S16.

Finally, based on the observed dose-dependent growth inhibition linked to compound **8** treatments in several T-ALL cell lines, we investigated biological processes downstream to **8**-dependent Notch signaling inhibition. We assessed, by flow cytometry analysis (FACS) after 7AAD staining, the effects of 36 hrs of treatment with compound **8** on cell cycle progression in DND41, KOPTK1 and TALL-1 cells. We observed a significant accumulation of cells in G1 phase following compound **8**-treatment in all the cell lines analysed (Figs 7a and S10). Concomitantly, we detected a substantial decreased expression of proteins involved in G1-S checkpoint progression, Cyclin D3 and the related kinases CDK4, accompanied by increased expression of the cyclin-dependent kinase inhibitor p27 which act as an inhibitor of G1-S transition (Fig. 7b). These data are consistent with the G1 cell cycle arrest previously observed after Notch1 pharmacological blockage by GSI treatment in Notch-driven T-cell leukaemia^32, 41^. In addition, we detected, by FACS analysis after Annexin V and 7AAD staining, a significant induction of apoptosis in human T-ALL cell lines after treatment with molecule **8** (Fig. 7c and S11). These data were further confirmed by the increased levels of the cleaved poly ADP-ribose polymerase PARP (Fig. 7d).

Notably, DAPT treatment, utilized as a positive control, was not able to affect cell cycle progression or apoptosis rate in T-ALL cells analysed (Fig. S12). This observation is in line with the GSI-dependent marked growth arrest previously reported in long terms of exposures (3–7 days) and not described in short terms treatments in T-ALL contexts^3, 42, 43^.

**Figure 8:**
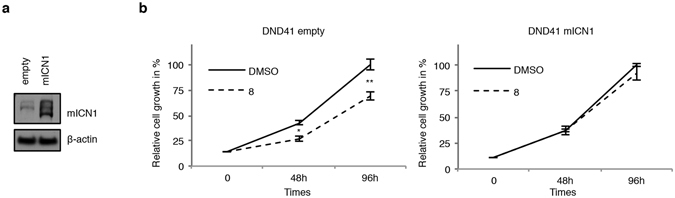
Compound **8** impairs cell growth through Notch inhibition. (**a**) Western blot analysis on GFP-sorted DND41 cells transduced with the mICN1 or with the empty control retroviruses by using antibodies against C-terminal domain of Notch1 and β-actin. (**b**) Effect of compound **8** treatment on the growth of GFP-sorted DND41 transduced with mICN1 or with the empty retroviruses for the times indicated. The relative cell number in percentage at each time point represents the mean value of triplicates normalized to the cell number at 96 hrs of DMSO treatment. Bars indicate S.D. *P < 0.05; **P < 0.01. Uncropped Western blots related to this figure are displayed in Fig. S17.

Finally, to further evaluate whether **8**-induced anti-growth effects in T-ALL are via Notch signaling inhibition, we treated with compound **8** DND41 cells transduced with retroviruses encoding murine N1ICD (mICN1) or with the relative empty retroviral vector (empty). Strength of transduction was evaluated by western blot against N1ICD (Fig. 8a). mICN1 enforced expression partially rescued the anti-proliferative effects induced by compound **8** in DND41 cells when compared to the control cells (Fig. 8b).

## Discussion

Increasing evidence indicates that the selective targeting of signaling pathways controlling Cancer Stem Cells (CSCs) self-behavior is a promising strategy to improve the effectiveness of the conventional anti-cancer chemo- and radio-therapies.

In the last few years, Notch signaling has been demonstrated to be essential in CSCs maintenance and self-renewal in several human tumors including T-ALL^44, 45^, thus providing the rationale for developing novel Notch inibitor-based therapies to prevent drug-resistance, recurrence and metastasis in solid and hematologic cancers, characterized by deregulated Notch signaling^15^. Current clinical investigations with regard to Notch inhibition in cancer are mostly focused on small molecules with GSI activity or on monoclonal antibodies (mABs) targeting specific Notch receptors and ligands^46, 47^, but although favourable outcomes are expected, both approaches show drawbacks that limit their widespread therapeutic use. Actually, GSIs are not Notch selective as they block the cleavage of a wide variety of trans-membrane proteins^48^. In addition, not all T-ALL harbouring Notch1 activating mutations are sensitive to GSI^36, 43^ and the treatment with GSIs often causes serious adverse side effects in patients^49, 50^. On the other hand, although mAbs have highly specific target selectivity, they show poor tissue and cell membrane penetration and mostly act on target antigens secreted by or expressed on the surface of tumor cells. Given the number of Notch deregulated signaling-related diseases and cancers, other than T-ALL leukemia, there is an urgent need to develop novel efficient and safe Notch-blocking therapies.

Following a DORS approach *in silico*, we identify here the 2′,3,4,4′-tetrahydroxychalcone (**C**) as a profitable Notch signaling inhibiting agent. Consistently with previous reports showing anti-proliferative properties of chalcones against numerous cancer cells^51, 52^, including acute lymphoblastic leukemia^53, 54^, we found a strict correlation between the **C**-induced Notch signaling blockage and the relative cellular response. Indeed, we found that short time exposure to molecule **C** substantially suppresses in a dose-dependent manner Notch1 activation and cell growth in human T-ALL cell lines exerting its biochemical and biological activities starting from concentration between 5 and 10 μM.

Accordingly, the chalcone scaffold emerged as a valuable tool for the design and synthesis of novel chalcone derivatives endowed with higher potency and most suitable pharmacological properties than the hit compound **C**. Twenty-six chalcones were synthesized and tested *in vitro*, highlighting the molecule **8** as the most potent Notch blocking agent of the series. Compound **8**, as well DAPT, suppresses endogenous Notch signaling activity in a short term treatment and in a low micro-molar range of exposure, as revealed by the decrease of the levels of the Notch activated domain and of the expression of several known Notch target genes in different human T-ALL cell models. Notably, we demonstrated that compound **8** does not interfere with the GS or ADAM activity, nor, at least in DND41 cells, with the early phases of receptor maturation process ending with the cell-surface localization of Notch receptor, thus encouraging future in-depth investigations to address its molecular mechanism of action.

Previous studies demonstrated that deregulated Notch signaling activation is linked to the uncontrolled growth of T-ALL cells and, consistently Notch inhibition by long term GSI treatments results in cell cycle arrest and apoptosis of GSI-sensitive cell lines^3, 42, 43^.

Here, we show that Notch inhibition, induced by short time exposure to chalcone–derived molecule **8** at low micro molar concentrations, results in anti-proliferative and pro-apoptotic effects in several human T-ALL cells, which were reverted by N1ICD overexpression, thus demonstrating that Notch1 inhibition is responsible, at least in part, of the growth inhibitory effect.

Notably, the data presented in our work suggest that compound **8** and the GSI DAPT are not pharmacologically equivalent and display significant differences in Notch inhibition.

In DND41, KOPTK1 and TALL-1 cells, short term exposures to compound **8** arrested G1/S cell-cycle progression further supported by the reduction of Cyclin D3 and CDK4 levels, both known to be regulated by Notch signaling to promote transition through the cell cycle during T-ALL leukemogenesis^41^. Moreover, in keeping with Dohda and colleagues data, which demonstrated that deregulated Notch signaling in T-ALL promotes cell cycle progression by maintaining low levels of expression of the anti-proliferative p27^55^, we observed that **8**-induced G1 cell cycle arrest in DND41, KOPTK1 and TALL-1 cells combined with increased expression levels of p27, further supporting the compound **8** anti-cancer activity leading to apoptosis of human T-ALL cells.

In summary, our results propose the chalcone derivative **8** as a novel and potent Notch inhibitor endowed with a naturally occurring scaffold, and provide novel guidelines for future development of chalcone-based bioactive Notch blocking agents, useful in the treatment of T-ALL and other cancers characterized by activated Notch signaling as single agents, or in combination with standard anti-cancer drugs.

## Methods

### Cheminformatics

A clustering algorithm adapted from that developed by Stahl and Mauser^28^ was generated in *Python* language by using the OpenEye Python Toolkit (OpenEye Toolkits 2015. OpenEye Scientific Software, Santa Fe, NM. http://www.eyesopen.com). The algorithm was then used to cluster the *in house* library of natural products and their derivatives by following a DORS approach. In particular, a cutoff of 0.6 was used for Tanimoto-based comparison of maccs166 type fingerprints, while a cutoff of 0.8 was used for the Raymond score^56^ during the analysis of maximum common substructures^28^.

### Source and characterization of natural compounds A – H

All the tested compounds **A–H** are known structures, which belong to our *in house* library of natural products. Chemical identity of compounds **A–H** was assessed by re-running NMR experiments, which proved to be in agreement with the literature data reported below for each compound. The purity of compounds **A**, **B**, **E** and **F**, checked by reversed-phase HPLC under the chromatographic conditions reported below, was always higher than 95%. Compound **A** (taxifolin or (2R,3R)-2-(3,4-dihydroxyphenyl)-3,5,7-trihydroxy-2,3-dihydrochromen-4-one) showed NMR spectra identical to the literature^57^. Compound **B** (4-hydroxy-isocordoin or (E)-1-[2,4-dihydroxy-3-(3-methylbut-2-enyl)phenyl]-3-(4-hydroxyphenyl)prop-2-en-1-one) showed NMR spectra identical to the literature^58^. Compound **C** (butein or (E)-1-(2,4-dihydroxyphenyl)-3-(3,4-dihydroxyphenyl)prop-2-en-1-one)^59^ was purchased from Sigma-Aldrich (487-52-5), and used without further modification. Compound **D** (xanthotoxin: 9-methoxyfuro[3,2-g]chromen-7-one)^60, 61^ was purchased from Sigma-Aldrich (298-81-7), and used without further modification. Compound **E** (columbianetin or (8R)-8-(2-hydroxypropan-2-yl)-8,9-dihydrofuro[2,3-h]chromen-2-one) showed NMR spectra identical to the literature^62^. Compound **F** (1-hydroxy-7-methoxy-9H-xanthen-9-one) showed NMR spectra identical to the literature^63^. Compound **G** (usnic acid or 2,6-diacetyl-7,9-dihydroxy-8,9b-dimethyldibenzofuran-1,3-dione)^64^ was purchased from Sigma-Aldrich (7562-61-0), and used without further modification. Compound **H** (galangin or 3,5,7-trihydroxy-2-phenylchromen-4-one)^65^ was purchased from Sigma-Aldrich (548-83-4), and used without further modification.

### Chemistry

All reagents were commercial and were used without further purification. Chromatography was carried out on silica gel (230–400 mesh). All reactions were monitored by thin-layer chromatography (TLC), and silica gel plates with fluorescence F254 were used. Purity of natural products, and the characterization of synthetic chalcones are reported in the Supplementary Materials section.

#### General procedure for the protection of differently substituted 3- or 4-hydroxybenzaldehydes as tetrahydropyranyl ethers

A differently substituted 3- or 4- hydroxybenzaldehyde (16 mmol) and pyridinium *p*-toluenesulfonate (0.40 mmol) (Sigma-Aldrich 232238) were dissolved in CH_2_Cl_2_ (dichloromethane) (75 ml) and 3,4-dihydro**-**α**-**pyran (2.23 ml, 0.05 mmol) (Sigma-Aldrich 37350) in CH_2_Cl_2_ (75 ml) was added dropwise. The reaction mixture was stirred at room temperature for 24 hrs, then washed with water, dried over Na_2_SO_4_ and evaporated *in vacuo*. The residue was purified by silica gel flash chromatography with *n*-Hexane: EtOAc = 9: 1 (v/v) as eluent, to obtain the corresponding tetrahydropyranyloxybenzaldehyde.

#### General procedure for the protection of differently substituted 4′-hydroxyacetophenones as tetrahydropyranyl ethers

A differently substituted 4′-hydroxyacetophenone (16 mmol) and pyridinium *p*-toluenesulfonate (0.40 mmol) were dissolved in CH_2_Cl_2_ (75 ml) and 3,4-dihydro**-**α**-**pyran (2.23 ml, 0.05 mmol) in CH_2_Cl_2_ (75 ml) was added dropwise. The reaction mixture was stirred at room temperature for 24 hrs, then washed with water, dried on Na_2_SO_4_ and evaporated *in vacuo*. The residue was purified by silica gel flash chromatography with *n*-Hexane: EtOAc = 9: 1 (v/v) as eluent, to obtain the corresponding tetrahydropyranyloxyacetophenone.

#### General procedure for the synthesis of chalcones

A solution of a suitable acetophenone (0.01 mol) and a suitable benzaldehyde (0.01 mol) were dissolved in EtOH (ethanol) and partially dehydrated barium hydroxide octahydrate (0.01 mol) was slowly added. The reaction mixture was stirred for 24 hrs at room temperature and then concentrated *in vacuo*. After water had been added, the mixture was neutralized with 2 M HCl and extracted with EtOAc (ethyl acetate). The organic layer was collected, dried over Na_2_SO_4_ and evaporated *in vacuo*. The residue was purified by silica gel flash chromatography with *n*-Hexane: EtOAc = 9: 1 (v/v) as eluent, to afford the corresponding chalcone **1–4, 9–10, 12–13, 5a-8a, 11a, 14a–17a**.

#### General procedure for the deprotection of chalcones

Protected chalcone **5a, 7a–8a, 11a, 14a–17a** (0.25 mmol) and *p*-toluenesulfonic acid (0.03 mmol) were dissolved in MeOH (20 ml). The reaction mixture was stirred for 24 hrs at room temperature and then evaporated in vacuo. After water had been added, the mixture was neutralized with 5% NaHCO_3_ and extracted with EtOAc. The organic layer was collected, dried over Na_2_SO_4_ and evaporated in vacuo. Purification of the residue via silica gel flash chromatography (n-Hexane: EtOAc = 9: 1 (v/v)) afforded the corresponding chalcone **5, 7–8, 11, 14–17**.

#### Catalytic hydrogenation of **8**

After two vacuum/nitrogen cycles to replace the air inside the reaction tube, compound **8** (100 mg, 0.37 mmol) and 10% Pd/C (98 mg) in dry EtOAc (10 mL) were vigorously stirred at room temperature under 1 atm of H_2_ for 24 hrs. The reaction mixture was filtered through a membrane filter (Millipore, Millex-LH, 0.45 mm) and the filtrate was concentrated giving compound **8b** in quantitative yield.

### Cell lines and treatments

DND41, KOPTK1 cells were cultured in RPMI-1640 (Gibco, Carlsbad, CA, USA) containing 10% fetal bovine serum. TALL-1 cells were maintained in RPMI-1640 (Gibco, Carlsbad, CA, USA) containing 20% fetal bovine serum. HEK and HaCat cells were cultured in Dulbecco’s modified Eagle’s medium (Gibco, Carlsbad, CA, USA) containing 10% fetal bovine serum. Cells were treated with: DAPT (565770; Calbiochem, Darmstadt, Germany), GI254023X (SML0789, Sigma-Aldrich, St Louis, MO, USA), compounds of natural origins **A**–**H** or the synthetics molecules **1–17** for the indicated concentrations and times of exposure.

### Immunoblot analysis

Protein extracts preparation was described elsewhere^66^. Blots were incubated with primary antibodies against: Notch1Val1744 (2421; Cell Signaling Technology, Beverly, MA, USA), Notch3 (2889; Cell Signaling Technology, Beverly, MA, USA), Notch1 (sc-6014-R; Santa Cruz Biotechnology, Santa Cruz, CA, USA), Cyclin D3 (sc-182; Santa Cruz Biotechnology, Santa Cruz, CA, USA), CDK4 (sc-260; Santa Cruz Biotechnology, Santa Cruz, CA, USA), p27 (3688; Cell Signaling Technology, Beverly, MA, USA), PARP (9542; Cell Signaling Technology, Beverly, MA, USA) and β-actin (sc-47778; Santa Cruz Biotechnology, Santa Cruz, CA, USA); followed by hybridization with Antibodies HRP conjugated: anti‐rabbit (sc-2004; Santa Cruz Biotechnology, Santa Cruz, CA, USA), or anti-mouse (sc-2005; Santa Cruz Biotechnology, Santa Cruz, CA, USA).

### Real-time PCR

Total RNA was extracted from cells using TRIzol (15596018; Invitrogen, Carlsbad, CA, USA) as described previously^67^. 100–1.000 ng of total RNA was reverse transcribed using High Capacity cDNA Reverse Transcription Kit (4368814; Applied Biosystems, Foster City, CA, USA) according to the manufacturer’s protocol. Real-time PCR was performed using the ViiA™ 7 Real-Time PCR System (Applied Biosystems, Foster City, CA, USA). Taqman Gene Expression Master Mix and Taqman Gene Expression Assays for HES1 (Hs00172878_m1), cMYC (Hs00905030_m1), DELTEX1 (Hs01092201_m1), NOTCH3 (Hs00166432_m1) and GAPDH (Hs02758991_g1) were purchased from Applied Biosystems, Foster City, CA, USA. Relative quantification was carried out using the comparative ΔΔ*C*t method. GAPDH expression was used to normalize mRNA levels.

### Statistical analysis

Mean values and standard deviations were calculated to estimate the degree of data variation, as detailed in the figure legends. The results were analyzed by using two-tailed Student’s *t*-test to calculate the statistical data significance.

## Electronic supplementary material

Supplementary Information

## References

[1] Liu J, Sato C, Cerletti M, Wagers A (2010). Notch signaling in the regulation of stem cell self-renewal and differentiation. Current topics in developmental biology.

[2] Palermo R, Checquolo S, Bellavia D, Talora C, Screpanti I (2014). The molecular basis of notch signaling regulation: a complex simplicity. Current molecular medicine.

[3] Weng AP (2004). Activating mutations of NOTCH1 in human T cell acute lymphoblastic leukemia. Science.

[4] Weng AP (2006). c-Myc is an important direct target of Notch1 in T-cell acute lymphoblastic leukemia/lymphoma. Genes & development.

[5] Purow BW (2008). Notch-1 regulates transcription of the epidermal growth factor receptor through p53. Carcinogenesis.

[6] Kumar V (2014). Notch and NF-kB signaling pathways regulate miR-223/FBXW7 axis in T-cell acute lymphoblastic leukemia. Leukemia.

[7] Ronchini C, Capobianco AJ (2001). Induction of cyclin D1 transcription and CDK2 activity by Notch(ic): implication for cell cycle disruption in transformation by Notch(ic). Molecular and cellular biology.

[8] Weijzen S (2002). Activation of Notch-1 signaling maintains the neoplastic phenotype in human Ras-transformed cells. Nature medicine.

[9] Vacca A (2006). Notch3 and pre-TCR interaction unveils distinct NF-kappaB pathways in T-cell development and leukemia. The EMBO journal.

[10] Talora C (2006). Cross talk among Notch3, pre-TCR, and Tal1 in T-cell development and leukemogenesis. Blood.

[11] Franciosa G (2016). Prolyl-isomerase Pin1 controls Notch3 protein expression and regulates T-ALL progression. Oncogene.

[12] Giambra V (2012). NOTCH1 promotes T cell leukemia-initiating activity by RUNX-mediated regulation of PKC-theta and reactive oxygen species. Nature medicine.

[13] Oudot C (2008). Prognostic factors for leukemic induction failure in children with acute lymphoblastic leukemia and outcome after salvage therapy: the FRALLE 93 study. J Clin Oncol.

[14] Pui CH, Robison LL, Look AT (2008). Acute lymphoblastic leukaemia. Lancet.

[15] Takebe N, Nguyen D, Yang SX (2014). Targeting notch signaling pathway in cancer: clinical development advances and challenges. Pharmacology & therapeutics.

[16] Deangelo DJ (2006). A phase I clinical trial of the notch inhibitor MK-0752 in patients with T-cell acute lymphoblastic leukemia/lymphoma (T-ALL) and other leukemias. J Clin Oncol.

[17] Mori M, Supuran CT (2015). Editorial: Challenging Organic Syntheses and Pharmacological Applications of Natural Products and their Derivatives. Current pharmaceutical design.

[18] Mori M, Supuran CT (2016). Challenging Organic Syntheses and Pharmacological Applications of Natural Products and their Derivatives - Part II. Current pharmaceutical design.

[19] Harvey AL (2008). Natural products in drug discovery. Drug discovery today.

[20] Harvey AL, Edrada-Ebel R, Quinn RJ (2015). The re-emergence of natural products for drug discovery in the genomics era. Nature reviews. Drug discovery.

[21] Koduru S, Kumar R, Srinivasan S, Evers MB, Damodaran C (2010). Notch-1 inhibition by Withaferin-A: a therapeutic target against colon carcinogenesis. Molecular cancer therapeutics.

[22] Wang Z, Zhang Y, Banerjee S, Li Y, Sarkar FH (2006). Notch-1 down-regulation by curcumin is associated with the inhibition of cell growth and the induction of apoptosis in pancreatic cancer cells. Cancer.

[23] Zhao M, Tang SN, Marsh JL, Shankar S, Srivastava RK (2013). Ellagic acid inhibits human pancreatic cancer growth in Balb c nude mice. Cancer letters.

[24] Mascarello A (2013). Discovery of Mycobacterium tuberculosis protein tyrosine phosphatase B (PtpB) inhibitors from natural products. PloS one.

[25] Cirigliano, A. *et al*. Yeast as a tool to select inhibitors of the cullin deneddylating enzyme Csn5. *Journal of enzyme inhibition and medicinal chemistry*, Papers in Press, published as 2010.3109/14756366.14752016.11160901, doi:10.3109/14756366.2016.1160901 (2016). 10.3109/14756366.2016.1160901 27028668

[26] Infante P (2015). Gli1/DNA interaction is a druggable target for Hedgehog-dependent tumors. The EMBO journal.

[27] Infante, P. *et al*. Inhibition of Hedgehog-dependent tumors and cancer stem cells by a newly identified naturally occurring chemotype. *Cell Death and Disease* Papers in Press, published as 2010.1038/cddis.2016.2195, doi:10.1038/cddis.2016.195 (2016). 10.1038/cddis.2016.195 PMC5059851 27899820

[28] Stahl M, Mauser H (2005). Database clustering with a combination of fingerprint and maximum common substructure methods. Journal of chemical information and modeling.

[29] Schweigert N, Zehnder AJ, Eggen RI (2001). Chemical properties of catechols and their molecular modes of toxic action in cells, from microorganisms to mammals. Environmental microbiology.

[30] Iovine V, Mori M, Calcaterra A, Berardozzi S, Botta B (2016). One Hundred Faces of Cyclopamine. Current pharmaceutical design.

[31] Congreve M, Carr R, Murray C, Jhoti H (2003). A ‘rule of three’ for fragment-based lead discovery?. Drug discovery today.

[32] Rao SS (2009). Inhibition of NOTCH signaling by gamma secretase inhibitor engages the RB pathway and elicits cell cycle exit in T-cell acute lymphoblastic leukemia cells. Cancer research.

[33] Sogawa S (1994). Protective effects of hydroxychalcones on free radical-induced cell damage. Biological & pharmaceutical bulletin.

[34] Kopan R, Schroeter EH, Weintraub H, Nye JS (1996). Signal transduction by activated mNotch: importance of proteolytic processing and its regulation by the extracellular domain. Proceedings of the National Academy of Sciences of the United States of America.

[35] Palomero T (2006). NOTCH1 directly regulates c-MYC and activates a feed-forward-loop transcriptional network promoting leukemic cell growth. Proceedings of the National Academy of Sciences of the United States of America.

[36] Palomero T (2007). Mutational loss of PTEN induces resistance to NOTCH1 inhibition in T-cell leukemia. Nature medicine.

[37] Bernasconi-Elias, P. *et al*. Characterization of activating mutations of NOTCH3 in T-cell acute lymphoblastic leukemia and anti-leukemic activity of NOTCH3 inhibitory antibodies. *Oncogene*, doi:10.1038/onc.2016.133 (2016). 10.1038/onc.2016.133 PMC5102827 27157619

[38] Jarriault S (1995). Signalling downstream of activated mammalian Notch. Nature.

[39] Yamamoto N (2001). Role of Deltex-1 as a transcriptional regulator downstream of the Notch receptor. The Journal of biological chemistry.

[40] Niimi H, Pardali K, Vanlandewijck M, Heldin CH, Moustakas A (2007). Notch signaling is necessary for epithelial growth arrest by TGF-beta. The Journal of cell biology.

[41] Joshi I (2009). Notch signaling mediates G1/S cell-cycle progression in T cells via cyclin D3 and its dependent kinases. Blood.

[42] Lewis HD (2007). Apoptosis in T cell acute lymphoblastic leukemia cells after cell cycle arrest induced by pharmacological inhibition of notch signaling. Chemistry & biology.

[43] O’Neil J (2007). FBW7 mutations in leukemic cells mediate NOTCH pathway activation and resistance to gamma-secretase inhibitors. The Journal of experimental medicine.

[44] Armstrong F (2009). NOTCH is a key regulator of human T-cell acute leukemia initiating cell activity. Blood.

[45] Tatarek J (2011). Notch1 inhibition targets the leukemia-initiating cells in a Tal1/Lmo2 mouse model of T-ALL. Blood.

[46] Andersson ER, Lendahl U (2014). Therapeutic modulation of Notch signalling–are we there yet?. Nature reviews. Drug discovery.

[47] Takebe N (2015). Targeting Notch, Hedgehog, and Wnt pathways in cancer stem cells: clinical update. Nature reviews. Clinical oncology.

[48] Bergmans BA, De Strooper B (2010). gamma-secretases: from cell biology to therapeutic strategies. The Lancet. Neurology.

[49] Milano J (2004). Modulation of notch processing by gamma-secretase inhibitors causes intestinal goblet cell metaplasia and induction of genes known to specify gut secretory lineage differentiation. Toxicological sciences: an official journal of the Society of Toxicology.

[50] Tolcher AW (2012). Phase I study of RO4929097, a gamma secretase inhibitor of Notch signaling, in patients with refractory metastatic or locally advanced solid tumors. J Clin Oncol.

[51] Wang Y, Chan FL, Chen S, Leung LK (2005). The plant polyphenol butein inhibits testosterone-induced proliferation in breast cancer cells expressing aromatase. Life sciences.

[52] Yit CC, Das NP (1994). Cytotoxic effect of butein on human colon adenocarcinoma cell proliferation. Cancer letters.

[53] Tang, Y. L. *et al*. Butein inhibits cell proliferation and induces cell cycle arrest in acute lymphoblastic leukemia via FOXO3a/p27kip1 pathway. *Oncotarget*, doi:10.18632/oncotarget.7624 (2016). 10.18632/oncotarget.7624 PMC4951317 26919107

[54] Moon DO, Kim MO, Lee JD, Choi YH, Kim GY (2009). Butein suppresses c-Myc-dependent transcription and Akt-dependent phosphorylation of hTERT in human leukemia cells. Cancer letters.

[55] Dohda T (2007). Notch signaling induces SKP2 expression and promotes reduction of p27Kip1 in T-cell acute lymphoblastic leukemia cell lines. Experimental cell research.

[56] Raymond JW, Gardiner EJ, Willett P (2002). Heuristics for similarity searching of chemical graphs using a maximum common edge subgraph algorithm. Journal of chemical information and computer sciences.

[57] Yang YN (2015). NMR spectroscopic method for the assignment of 3,5-dioxygenated aromatic rings in natural products. Journal of natural products.

[58] Delle Monache G (1974). New prenylated flavonoids of Cordoa piaca (Lonchocarpus sp.). Gazz. Chim. Ital..

[59] Chokchaisiri R (2009). Bioactive flavonoids of the flowers of Butea monosperma. Chemical & pharmaceutical bulletin.

[60] Compagnone R, Rodriguez MC (1993). Coumarins from Pilocarpus racemosus. Fitoterapia.

[61] Rashid MA, Gray AI, Waterman PG (1995). Myrtenyl acetate from Phebalium tuberculosum ssp. megaphyllum. Fitoterapia.

[62] Cuca LE MJ, Delle Monache F (1998). Constituyentes quimicos de Zanthoxylum monophyllum. Revista Colombiana de Quimica.

[63] Delle Monache F, Marquina Mac-Quhae M, Delle Monache G, Marini-Bettolo GB, Alves De Lima A (1983). Xanthones, xanthonolignoids and other constituents of the roots of vismia guaramirangae. Phytochemistry.

[64] Studzinska-Sroka E (2015). *In vitro* antimicrobial activity of extracts and compounds isolated from Cladonia uncialis. Natural product research.

[65] Zhong L (2012). Antimicrobial flavonoids from the twigs of Populus nigra x Populus deltoides. Natural product research.

[66] Aribi B, Zerizer S, Kabouche Z, Screpanti I (2016). & Palermo, R. Effect of Argania spinosa oil extract on proliferation and Notch1 and ERK1/2 signaling of T-cell acute lymphoblastic leukemia cell lines. Food and Agricultural Immunology.

[67] Cialfi S (2013). Glucocorticoid sensitivity of T-cell lymphoblastic leukemia/lymphoma is associated with glucocorticoid receptor-mediated inhibition of Notch1 expression. Leukemia.

