# Supplementary material for: Identification of a novel chalcone derivative that inhibits Notch signaling in T-cell acute lymphoblastic leukemia
Source: Sci Rep. 2017 May 19;7:2213. doi: 10.1038/s41598-017-02316-9 (PMC5438367; doi:10.1038/s41598-017-02316-9)
Supplement: Supplementary file 1 — Supplementary Information [file 41598_2017_2316_MOESM1_ESM.pdf]

Identification of a novel chalcone derivative that inhibits Notch signaling in T-cell acute lymphoblastic leukemia

Mattia Mori[1,5], Luca Tottone[2,5], Deborah Quaglio[3,5], Nadezda Zhdanovskaya[2], Cinzia Ingallina[3], Marisa Fusto[2], Francesca Ghirga[1], Giovanna Peruzzi[1], Maria Elisa Crestoni[3], Fabrizio Simeoni[2], Francesca Giulimondi[2], Claudio Talora[2], Bruno Botta[3,*], Isabella Screpanti[2,4,*], Rocco Palermo [1,*]

Affiliations:

[1]Center for Life Nano Science@Sapienza; Istituto Italiano di Tecnologia; Rome; 00161; Italy;

[2]Department of Molecular Medicine; Sapienza University of Rome; Rome; 00161; Italy;

[3]Department of Chemistry and Technology of Drugs; Sapienza University of Rome; Rome; 00185; Italy;

[4]Istituto Pasteur Fondazione Cenci Bolognetti; Sapienza University of Rome; Rome; 00161; Italy;

*Correspondence to: rocco.palermo@iit.it (R.P.); isabella.screpanti@uniroma1.it (I.S.); bruno.botta@uniroma1.it (B.B.);

[5]Co-first authors;

SUPPLEMENTARY FIGURES:

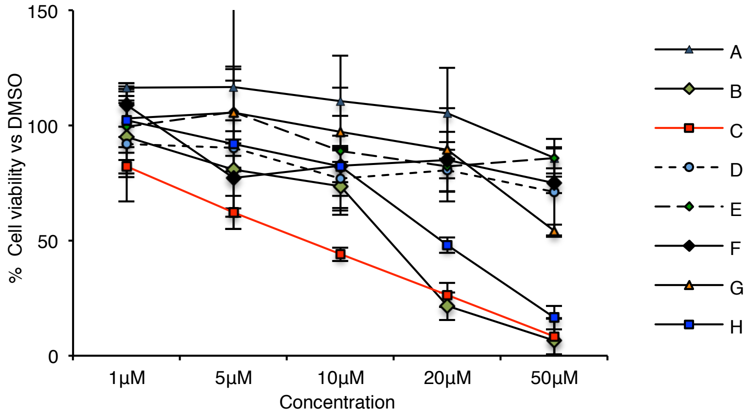

Fig. S1. Butein is the most powerful natural compound in inhibiting DND41 cell growth. Dose-response curves by MTS cell viability assay towards DND41 cells at 36 hrs incubation times with the 8 molecules (named **A**, **B**, **C**, **D**, **E**, **F**, **G** and **H**) representative of the *in house* library consisting of 1000 compounds. Data represent mean of values for triplicates ± S.D..

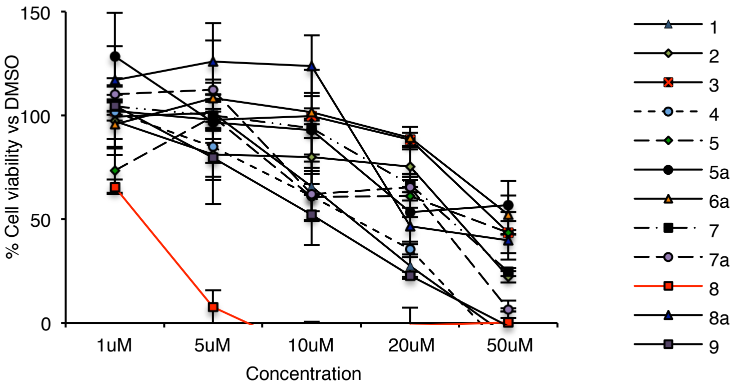

Fig. S2. Compound 8 is the most powerful C-derivative in inhibiting DND41 cell growth. Dose-response curves by MTS cell viability assay towards DND41 cells at 36 hrs incubation times with the twelve molecules C-derivative (named **1**, **2**, **3**, **4**, **5**, **5a**, **6a**, **7**, **7a**, **8**, **8a** and **9**). Data represent mean of values for triplicates ± S.D..

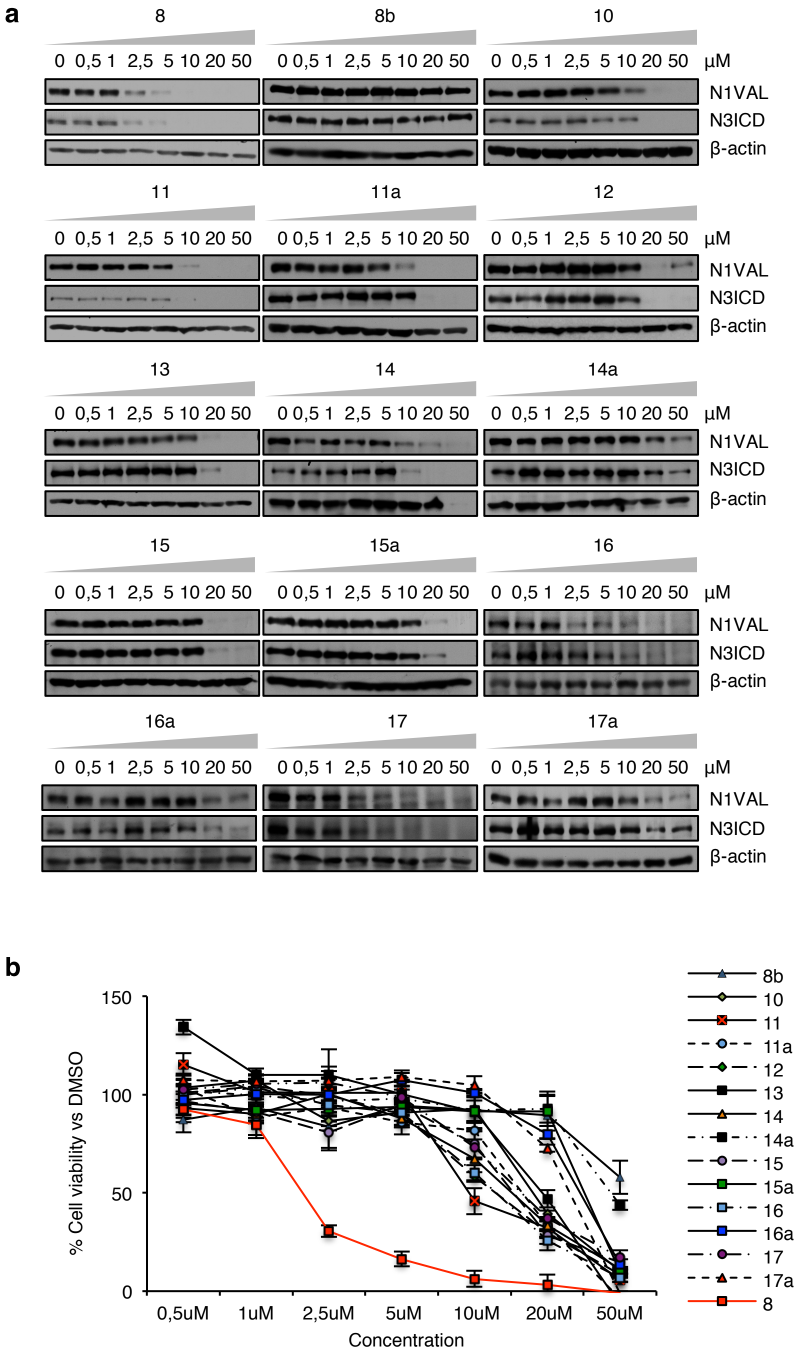

Fig. S3. Compound 8 derivatives exert lower bioactivity in DND41 cells, when compared to compound 8. (**a**) N1VAL, N3ICD and β-actin protein expression levels and (**b**) curves by MTS cell viability assay in DND41 T-ALL cell lines in response to 36 h of dose dependent manner exposure to **8** and fourteen molecules **8**-derivative (named **8b**, **10**, **11**, **11a**, **12**, **13**, **14**, **14a**, **15**, **15a**, **16**, **16a**, **17** and **17a**). Data represent mean of values for triplicates ± S.D..

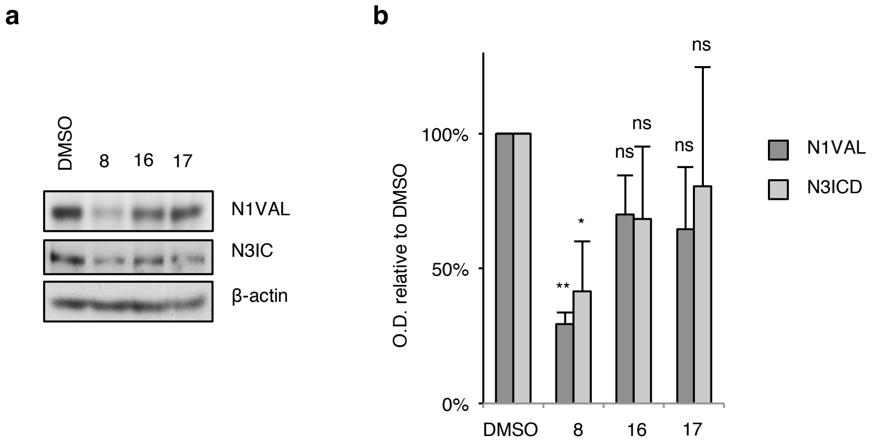

Fig. S4. Compound 8 shows the strongest Notch inhibitory potency if compared to its derivatives 16 and 17. (**a**) Representative western blots and (**b**) relatives optical densitometric quantifications (O.D.) of N1VAL and N3ICD protein expressions in DND41 cells in response to 36hrs of treatment with 2.5 μM of **8**, **16** or **17**. β-actin is used as loading control. Data in bar graphs are expressed as mean ± S.D. of three independent assays. *P<0.05; **P<0.01; ns not significant.

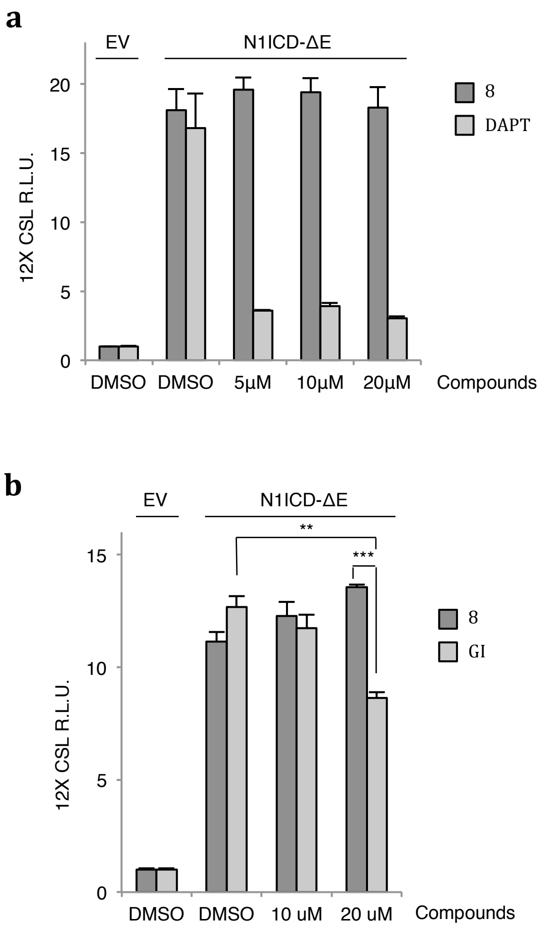

Fig. S5. Compound 8 does not act as a GSI. Luciferase assay performed on HEK 293T cells co-transfected with Notch-responsive 12X CSL-Luciferase reporter (12X CSL) in combination with the plasmid pCS2 Notch1 ΔEMV-6MT (N1ICD-ΔE) or with the empty vector (EV), and treated with different doses of (**a**) compound **8** or DAPT, and of (**b**) compound **8** or GI254023X (GI). Data represent mean of values for triplicates ± S.D.. **P<0.01; ***P<0.001.

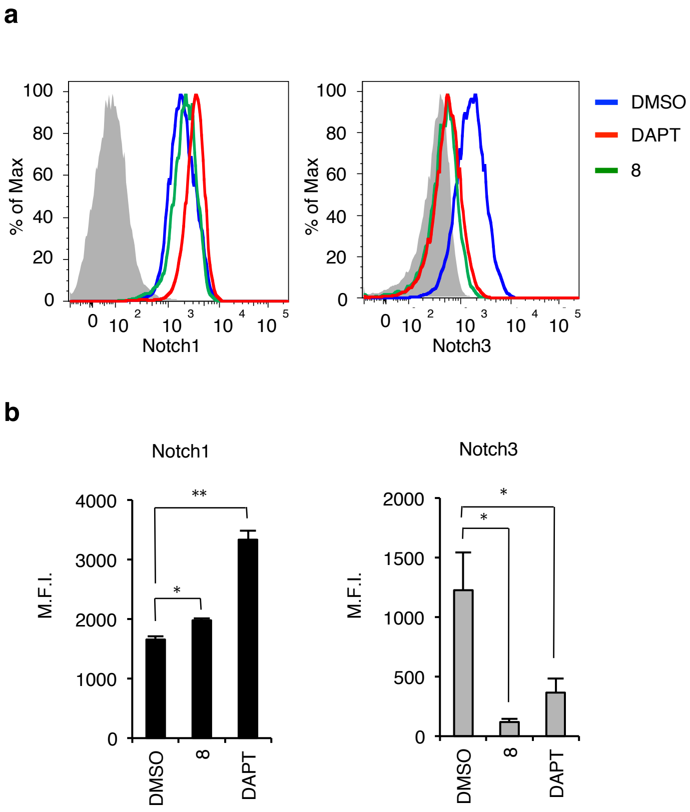

Fig. S6. Notch1 and Notch3 surface expression in DND41 cells treated with compound 8 or DAPT. (**a**) Notch1 and Notch3 surface expressions were investigated by flow cytometry analysis in DND41 cells treated for 36 hrs with 2.5 µM of compound **8** or DAPT or with the vehicle alone (DMSO). Solid grey histograms represent the isotype controls. Histogram plots shown in figure are representative of results obtained from three independent experiments. (**b**) Histograms show the mean of the ratio of Median Fluorecence intensity values (M.F.I.) between Notch1 and Notch3 surface expression levels respect to their isotype controls ± S.D.. *P<0.05; **P<0.01.

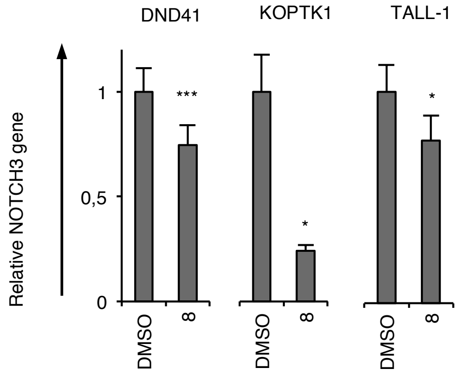

Fig. S7. Compound 8 decreases Notch3 gene expression in T-ALL cells. Relative NOTCH3 gene expression levels in response to 36hrs of treatment with 2.5 µM of molecule **8** in DND41, KOPTK1 and TALL-1 cells. Data represent mean values ± S.D. normalized to GAPDH of at least two independent experiments. *P<0.05; ***P<0.001.

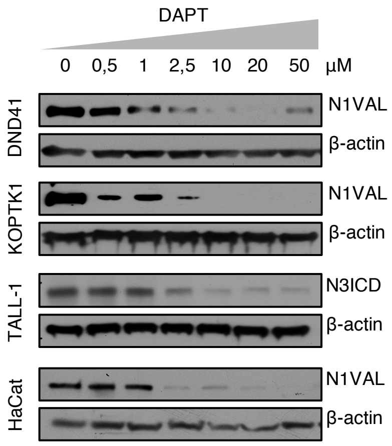

Fig. S8. Notch signaling activity modulation by DAPT. N1VAL, N3ICD and β-actin protein expression levels in response to 36 hrs dose dependent manner of DAPT exposures in DND41, KOPTK1, TALL-1 and HaCat cells.

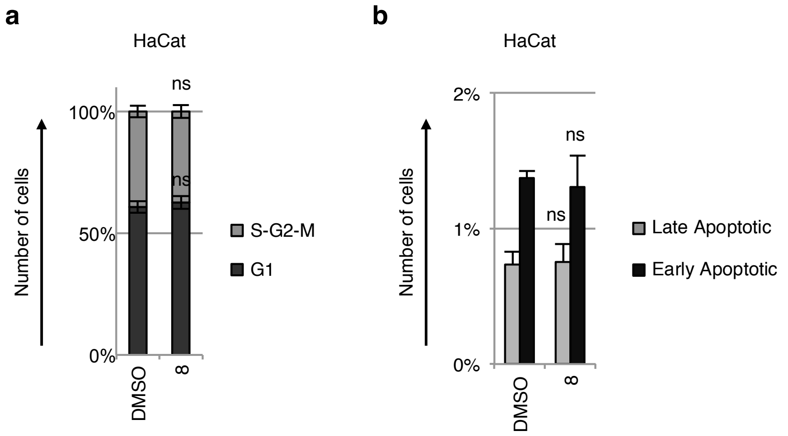

Fig. S9. Compound 8 treatment does not affect cell cycle and apoptosis in HaCat cells. HaCat cells were treated for 36 hrs with 2.5 μM of compound **8** or with vehicle alone (DMSO). Cell cycle progression was investigated by FACS analysis of DNA content after 7AAD staining. (**a**) Histograms show the mean of cell percentage ± S.D. in G1 versus S-G2-M phases of cell cycle triplicates. Apoptosis rate were investigated by FACS analysis after Annexin V and 7AAD staining. (**b**) Histograms show the mean of cell percentage ± S.D. of early and late apoptotic of triplicates. ns not significant.

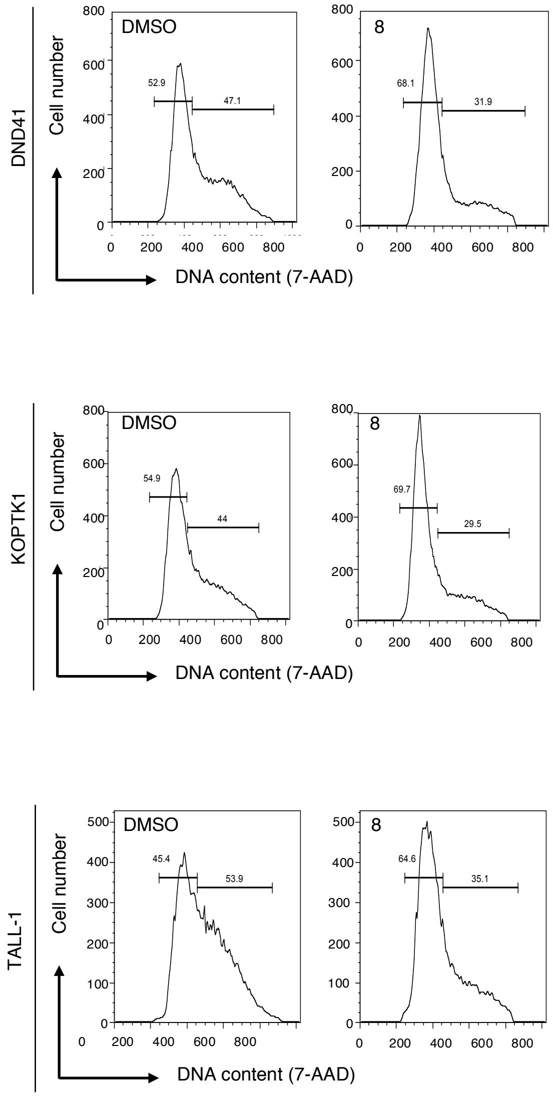

Fig. S10. Notch signaling inhibition with compound 8 impairs cell cycle progression in T-ALL cells. DND41, KOPTK1 and TALL-1 cells were treated for 36 hrs with compound **8** or vehicle alone (**DMSO**) with the doses indicated in the text. Cell cycles were investigated by flow cytometry analysis of DNA content after 7AAD staining. FACS plots shown in figure are representative of results obtained from four independent experiments.

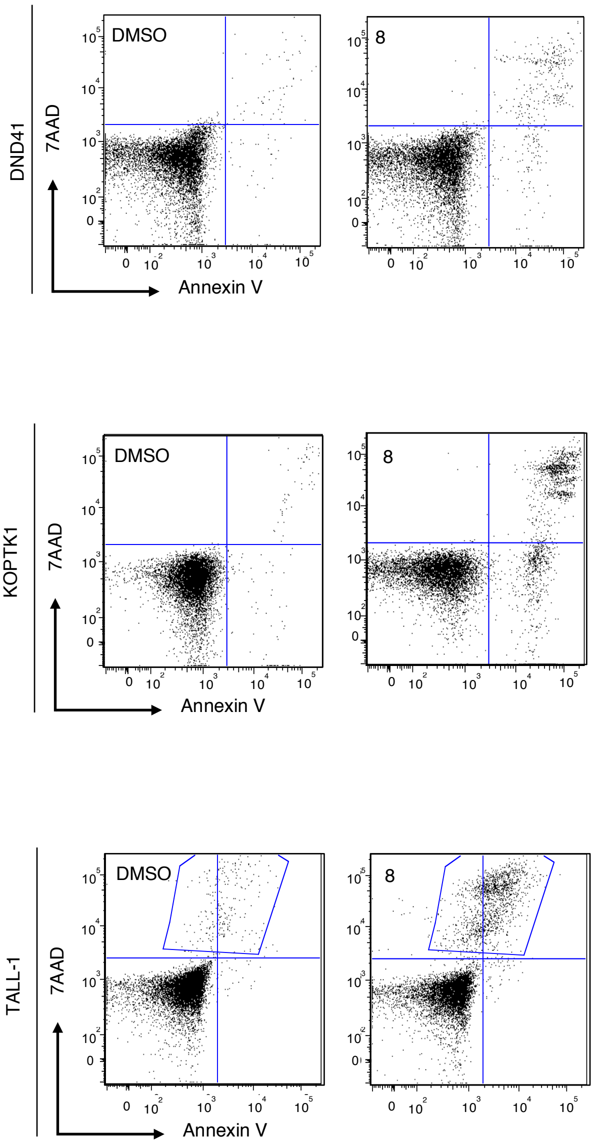

Fig. S11. Compound 8 treatment induces apoptosis in in T-ALL cells. DND41, KOPTK1 and TALL-1 cells were treated for 36 hrs with compound **8** or vehicle alone (DMSO) with the doses indicated in the text. Apoptosis rates were investigated by flow cytometry analysis after Annexin V and 7AAD staining. FACS plots shown in figure are representative of results obtained from at least four independent experiments.

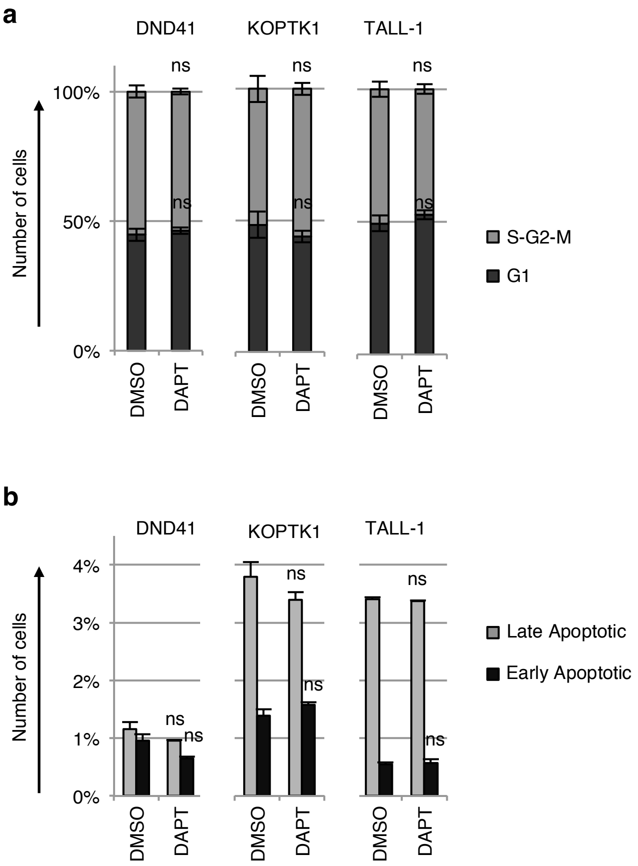

Fig. S12. Short times of DAPT treatment does not affects cell cycle and apoptosis in human T-ALL cells. DND41, KOPTK1 and TALL-1 cell lines were treated for 36 hrs with 2.5 μM of DAPT or with vehicle alone (DMSO). Cell cycle progression was investigated by FACS analysis of DNA content after 7AAD staining. (a) Histograms show the mean of cell percentage ± S.D. in G1 versus S-G2-M phases of cell cycle of triplicates. (b) Apoptosis rates were investigated by FACS analysis after Annexin V and 7AAD staining. (b) Histograms show the mean of cell percentage ± S.D. of early and late apoptotic of triplicates. ns not significant.

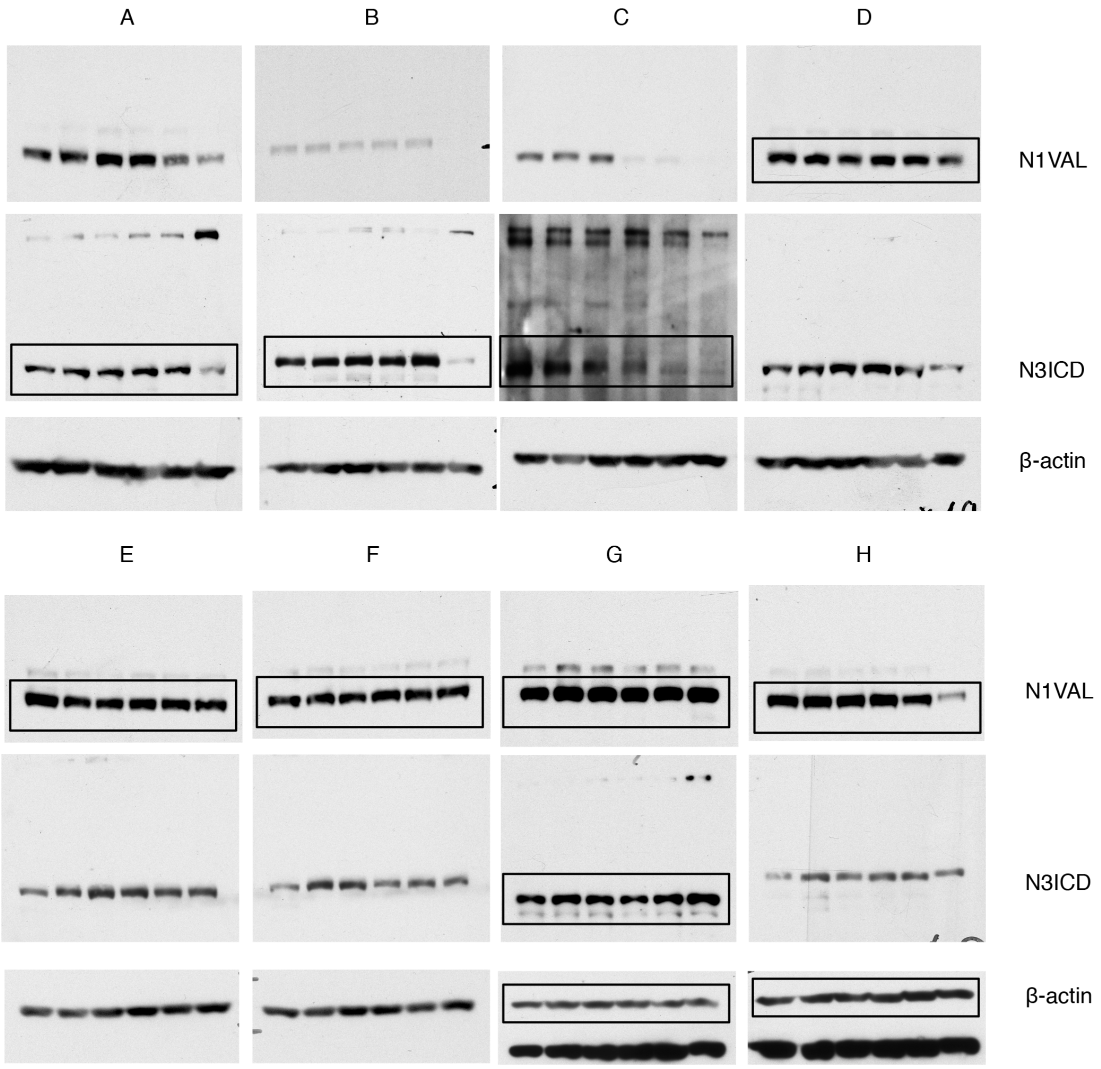

Fig. S13. Uncropped Western blots related to main Fig. 1.

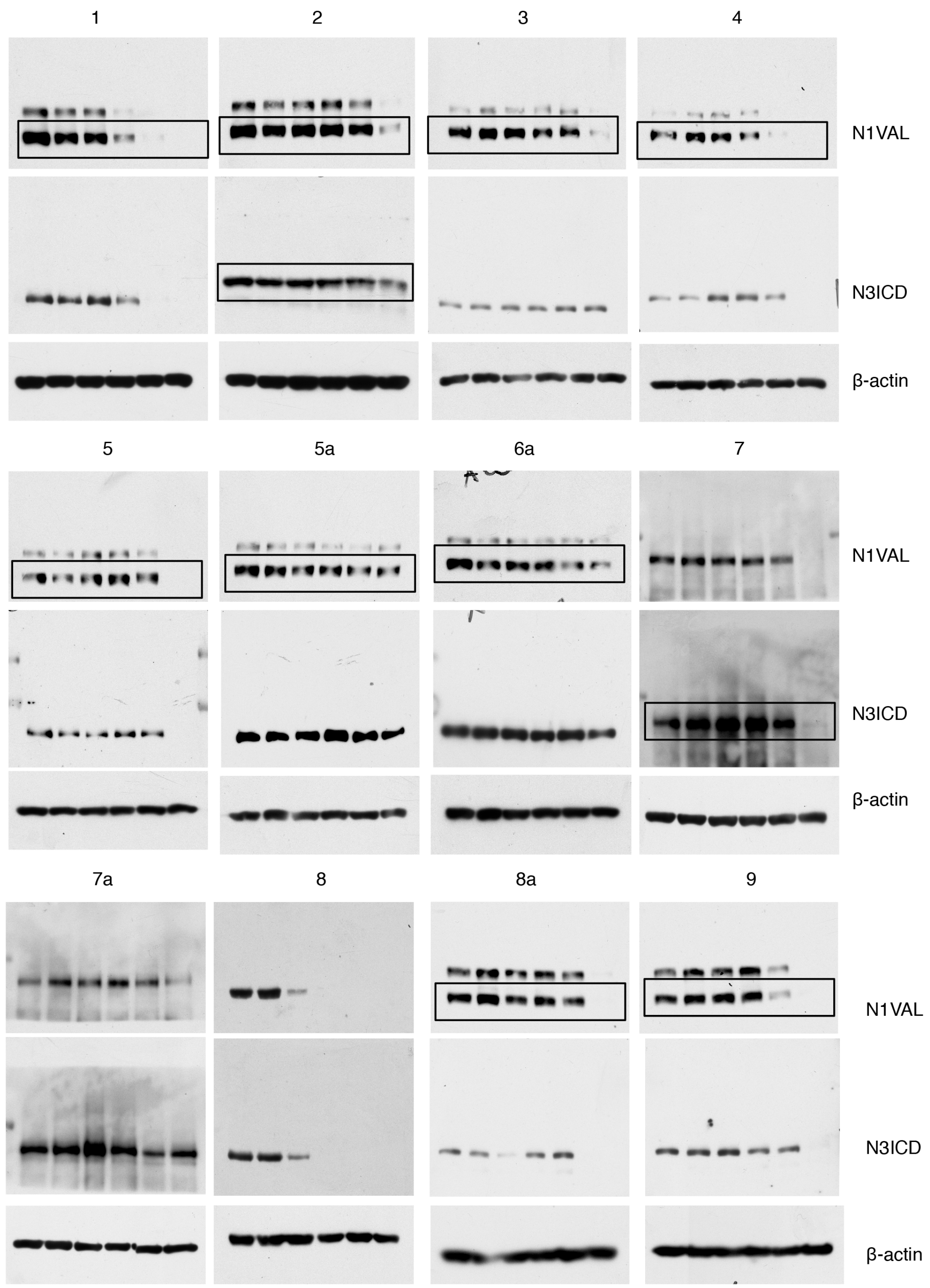

Fig. S14. Uncropped Western blots related to main Fig. 4.

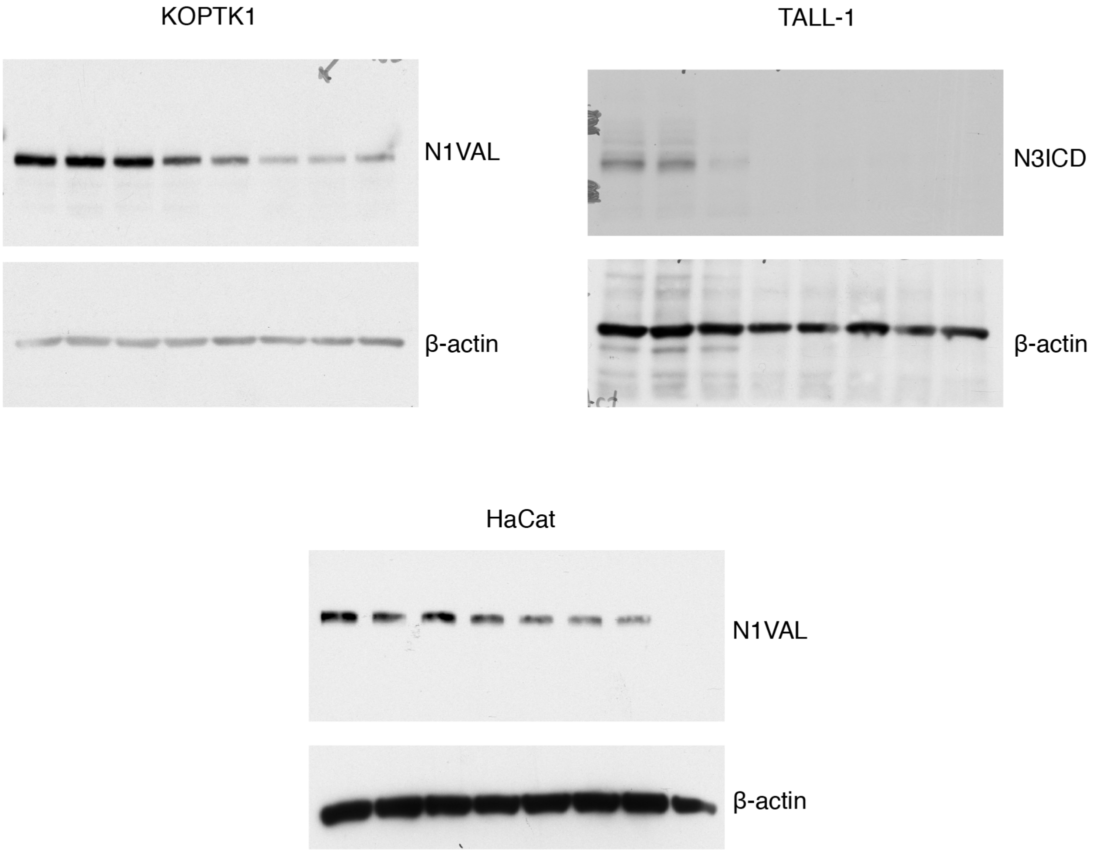

Fig. S15. Uncropped Western blots related to main Fig. 6.

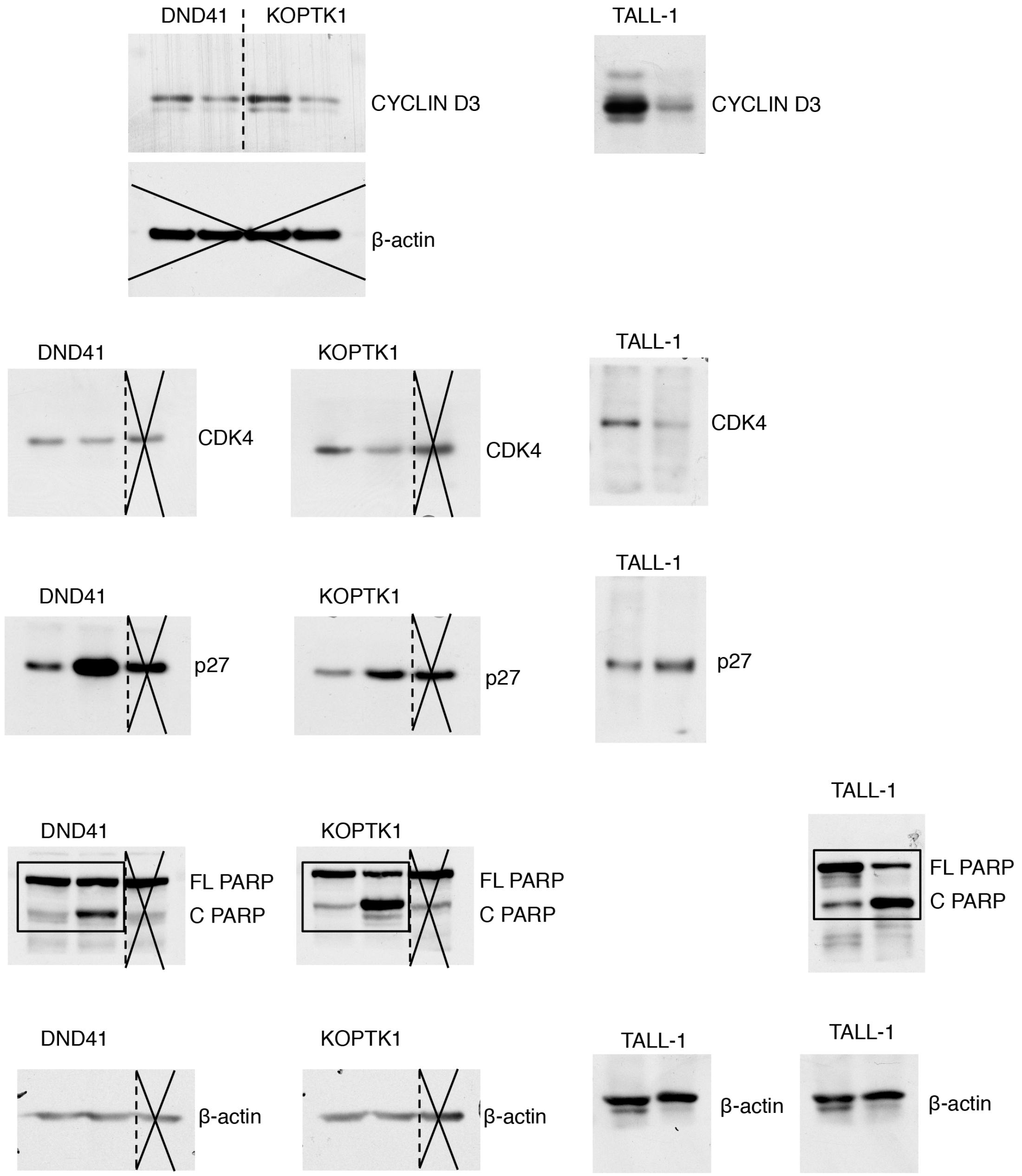

Fig. S16. Uncropped Western blots related to main Fig. 7.

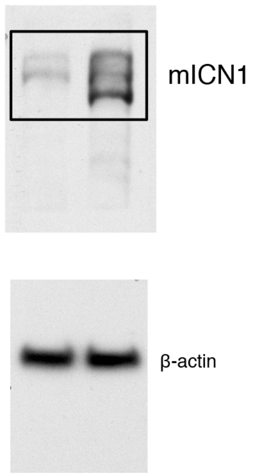

Fig. S17. Uncropped Western blots related to main Fig. 8.

SUPPLEMENTARY METHODS

General Experimental Methods

Melting points were taken in open capillaries on a Büchi Melting Point B-545 apparatus and are presented uncorrected. ^{1}H NMR and ^{13}C NMR spectra were recorded using a Bruker 400 Ultra Shield^{TM} spectrometer (operating at 400 MHz for ^{1}H and 100 MHz for ^{13}C) using tetramethylsilane (TMS) as internal standard. Chemical shifts are reported in parts per million (ppm). Multiplicities are reported as follows: singlet (s), doublet (d), triplet (t) and multiplet (m). Mass spectrometry was performed using a Thermo Finnigan LXQ linear ion trap mass spectrometer, equipped with an electrospray ionization (ESI) ion source bearing a steel needle. High resolution mass spectra (HRMS) were recorded on Bruker BioApex Fourier transform ion cyclotron resonance (FT-ICR) mass spectrometer.

2',3,4,4'-tetramethoxychalcone (**1**) Yellow solid (yield 85%); mp 71.9-73.3 °C. ^{1}H NMR (400 MHz, $CDCl_3$): δ 7.72 (H-6', 1H, d), δ 7.61 ($C_{\beta}H$, 1H, d, J=16.0 Hz), δ 7.35 ($C_{\alpha}H$-CO, 1H, d, J=16.0 Hz), δ 7.18 (H-6, 1H, dd, J=8.4 Hz e J=1.6 Hz), δ 7.11 (H-2, 1H, d, J=2.6 Hz), δ 6.87 (H-5, 1H, d, J=8.4 Hz), δ 6.56 (H-5', 1H, dd, J=8.4 Hz and J=2.0 Hz), δ 6.50 (H-3', 1H, d, J=2 Hz), δ 3.92 (OCH_3, 3H, s), δ 3.92 (OCH_3, 3H, s), δ 3.90 (OCH_3, 3H, s), δ 3.87 (OCH_3, 3H, s). ^{13}C NMR (100 MHz, $CDCl_3$) : δ 190.73, δ 163.94, δ 160.20, δ 150.97, δ 149.14, δ 142.43, δ 132.66, δ 128.44, δ 125.32, δ 122.61, δ 122.46, δ 111.12, δ 110.27, δ 105.09, δ 98.75, δ 55.97, δ 55.75, δ 55.53. HRMS (ESI- FT-ICR) m/z: $[M + H]^+$ calcd for $C_{19}H_{21}O_5$ 329.13852 (monoisotopic mass), found 329.13857; $[M + Na]^+$ calcd for $C_{19}H_{20}O_5Na$ 351.12029 (monoisotopic mass), found 351.11879.

2'-hydroxy-3,4,4'-trimethoxychalcone (**2**) Yellow solid (yield 50%); mp 157.8-158.3 °C. ^{1}H NMR (400 MHz, $CDCl_3$): δ 13.54 (OH, 1H, s), δ 7.85 ($C_{\beta}H$, 1H, d, J=15.6 Hz,), δ 7.86 (H-6', 1H, d), δ 7.44 ($C_{\alpha}H$-CO, 1H, d, J=16.0 Hz), δ 7.25 (H-6, 1H, dd, J=8.1 Hz and J=6 Hz), δ 7.16 (H-2, 1H, d, J=2 Hz), δ 6.91 (H-5, 1H, d, J=8.4 Hz) , δ 6.51-6.47 (H-3'and H-5', 2H, m), δ 3.97 (OCH_3, 3H, s), δ 3.94 (OCH_3, 3H, s), δ 3.86 (OCH_3, 3H, s). ^{13}C NMR (100 MHz, $CDCl_3$): δ 191.80, δ 166.67, δ 166.08, δ 151.61, δ 149.31, δ 144.59, δ 131.13, δ 127.81, δ 123.33, δ 118.05, δ 114.14, δ 111.18, δ 110.26, δ 107.66, δ 101.06, δ 56.03, δ 55.59, δ 53.42. HRMS (ESI- FT-ICR) m/z: $[M + H]^+$ calcd for $C_{18}H_{19}O_5$

315.12270 (monoisotopic mass), found 315.12313; [M + Na]$^+$ calcd for $C_{18}H_{18}O_5Na$ 377.10464 (monoisotopic mass), found 337.10489.

2'-hydroxy-4,4'-dimethoxychalcone (**3**) Yellow solid (yield 25%); mp 133.4-134.2 °C. ^{1}H NMR (400 MHz, $CDCl_3$): δ 13.56 (OH, 1H, s), δ 7.87 (C_βH, 1H, d, J=15.6 Hz,), δ 7.84 (H-6', 1H, d, J= 8 Hz), δ 7.62 (H-2 and H-6, 2H, d, J= 8.8 Hz), δ 7.47 (C_αH-CO, 1H, d, J= 15.6 Hz), δ 6.95 (H-3 and H-5, 2H, d, J= 8.8 Hz), δ 6.50-6.48 (H-3' and H-5', 2H, m); δ 3.87 (OCH_3, 3H, s); δ 3.86 (OCH_3, 3H, s). ^{13}C NMR (100 MHz, $CDCl_3$): δ 191.90, δ 166.64, δ 166.05, δ 161.82, δ 144.28, δ 131.12, δ 130.37, δ 127.56, δ 117.85, δ 114.48, δ 114.17, δ 107.63, δ 101.07, δ 55.59, δ 55.44. HRMS (ESI- FT-ICR) m/z: [M + H]$^+$ calcd for $C_{17}H_{17}O_4$ 285.11214 (monoisotopic mass), found 285.11213; [M + Na]$^+$ calcd for $C_{17}H_{16}O_4Na$ 307.09408 (monoisotopic mass), found 307.09413.

2',4,4'-trimethoxychalcone (**4**) Yellow solid (yield 12%); mp 87.2-88.2 °C. ^{1}H NMR (400 MHz, $(CD_3)_2CO$): δ 7.70 (H-2, H-6 and H-6', 3H, m), δ 7.62 (C_βH, 1H, d, J=15.6 Hz,), δ 7.53 (C_αH-CO, 1H, d, J= 15.6 Hz), δ 7.03 (H-3 and H-5 , 2H, m), δ 6.71 (H-3', 1H, d, J= 2 Hz); δ 6.66 (H-5', 1H, dd, J=8 Hz and J=2 Hz); δ 3.99 (OCH_3, 3H, s); δ 3.93 (OCH_3, 3H, s); δ 3.89 (OCH_3, 3H, s). ^{13}C NMR (100 MHz, $(CD_3)_2CO$): δ 189.08, δ 164.23, δ 161.45, δ 160.47, δ 140.87, δ 132.21, δ 131.73, δ 129.89, δ 128.13, δ 125.12, δ 122.33, δ 114.34, δ 113.22, δ 105.70, δ 98.34, δ 55.32, δ 55.06, δ 54.85. HRMS (ESI- FT-ICR) m/z: [M + H]$^+$ calcd for $C_{18}H_{19}O_4$ 299.12779 (monoisotopic mass), found 299.12822; [M + Na]$^+$ calcd for $C_{18}H_{18}O_4Na$ 321.10973 (monoisotopic mass), found 321.10938.

2'-hydroxy-4,4'-dimethoxy-3-(tetrahydropyran-2-yloxy)chalcone (**5a**) Yellow solid (yield 35%); mp 123.9-125.9 °C. ^{1}H NMR (400 MHz, $CDCl_3$): δ 13.55 (OH, 1H, s), δ 7.83 (C_βH, 1H, d, J=15 Hz), δ 7.82 (H-6', 1H, d, J=8 Hz), δ 7.47 (H-2, 1H, d, J=2 Hz), δ 7.42 (C_αH-CO, 1H, d, J=15.0 Hz), δ 7.28 (H-6, 1H, dd, J=8.4 Hz and J=1.6 Hz), δ 6.92 (H-5, 1H, d, J=8.4 Hz) , δ 6.49-6.47 (H-3'and H-5', 2H, m), δ 5.47 (H-1", 1H, m), δ 4.01 (H_α-5", 1H, m), δ 3.91 (OCH_3, 3H, s), δ 3.86 (OCH_3, 3H, s), δ 3.65 (H_β-5", 1H, m), δ 2.08-1.67 (H-2", H3" and H-4", 6H, m) . ^{13}C NMR (100 MHz, $CDCl_3$): δ 191.86, δ 166.64, δ 166.09, δ 148.94, δ 145.98, δ 144.34, δ 131.16, δ 128.51, δ 122.94, δ 118.48, δ 113.02, δ 110.61, δ 107.63, δ 101.11, δ 94.68, δ 62.94, δ 56.06, δ 55.58, δ 31.93, δ 30.89, δ 25.46, δ 19.76. HRMS (ESI- FT-ICR) m/z: [M + H]$^+$ calcd for $C_{22}H_{25}O_6$ 385.16456 (monoisotopic mass), found 385.16486; [M + Na]$^+$ calcd for $C_{22}H_{24}O_6Na$ 407.14651 (monoisotopic mass), found 407.14568.

2’,3-dihydroxy-4,4’-dimethoxychalcone (**5**) Yellow solid (quantitative yield); mp 161.6-164.0 °C. ^{1}H NMR (400 MHz, DMSO-d_6): δ 13.63 (OH, 1H, s), δ 9.30 (OH, 1H, s) δ 8.29 (H-6’, 1H, d, J=9.2 Hz), δ 7.82 (C_{β}H, 1H, d, J=15.2 Hz), δ 7.75 (C_{α}H-CO, 1H, d, J=16 Hz), δ 7.41 (H-2, 1H, d, J=1.6 Hz), δ 7.36 (H-6, 1H, dd, J=8.4 Hz and J=1.6 Hz), δ 7.04 (H-5, 1H, d, J=8.4 Hz) , δ 6.59 (H-5’, 1H, dd, J=9.2 Hz and J=2.4 Hz), δ 6.54 (H-3’, 1H, d, J=2.4 Hz), δ 3.88 (OCH_3, 6H, s). ^{13}C NMR (100 MHz, DMSO-d6): δ 192.28, δ 166.26, δ 166.20, δ 151.02, δ 147.19, δ145.22, δ 133.00, δ 128.01, δ 122.90, δ 118.88, δ 115.64, δ 114.40, δ 112.34, δ 107.72, δ 101.42, δ 56.19, δ 56.17. HRMS (ESI- FT-ICR) m/z: [M + H]$^+$ calcd for $C_{17}H_{17}O_5$ 301.10705 (monoisotopic mass), found 301.10709; [M + Na]$^+$ calcd for 323.08899 $C_{17}H_{16}O_5$Na 323.08899 (monoisotopic mass), found 323.08861.

2’-hydroxy-3,4-dimethoxy-4’-(tetrahydropyran-2-yloxy)chalcone (**6a**) Yellow solid (yield 18%); mp 116.3-118.6 °C. ^{1}H NMR (400 MHz, $CDCl_3$): δ 13.37 (OH, 1H, s), δ 7.85 (C_{β}H, 1H, d, J=15.2 Hz), δ 7.84 (H-6’, 1H, d, J=9.2 Hz), δ 7.44 (C_{α}H-CO, 1H, d, J=15.2 Hz), δ 7.25 (H-6, 1H, dd, J=8.4 Hz and J=1.6 Hz), δ 7.16 (H-2, 1H, d, J=1.6 Hz), δ 6.91 (H-5, 1H, d, J=8.4 Hz), δ 6.67 (H-3’, 1H, d, J=2.4 Hz), δ 6.60 (H-5’, 1H, dd, J=8.8 Hz and J=2.4 Hz), δ 5.51 (H-1”, 1H, t), δ 3.96 (OCH_3, 3H, s), δ 3.94 (OCH_3, 3H, s), δ 3.89-3.83 (H_{α}-5”, 1H, m), δ 3.66-3.61 (H_{β}-5”, 1H, m), δ 1.89-1.60 (H-2”, H3” and H-4”, 6H, m) .^{13}C NMR (100 MHz, $CDCl_3$): δ 191.95, δ 166.21, δ 163.53, δ 151.60, δ 149.31, δ144.64, δ 131.12, δ 127.82, δ 123.34, δ 118.10, δ 114.82, δ 111.19, δ 110.28, δ 108.45, δ 104.28, δ 96.17, δ 62.24, δ 56.03, δ 30.01, δ 25.00, δ 18.55. HRMS (ESI- FT-ICR) m/z: [M + H]$^+$ calcd for $C_{22}H_{25}O_6$ 385.16456 (monoisotopic mass), found 385.16512; [M + Na]$^+$ calcd for $C_{22}H_{24}O_6$Na 407.14651 (monoisotopic mass), found 407.14554.

2’-hydroxy-3,4’-dimethoxy-4-(tetrahydropyran-2-yloxy)chalcone (**7a**) Yellow solid (yield 20%); mp 90.5-93.0 °C. ^{1}H NMR (400 MHz, $CDCl_3$): δ 13.37 (OH, 1H, s), δ 7.84 (C_{β}H, 1H, d, J=15.2 Hz), δ 7.84 (H-6’, 1H, d, J=9.2 Hz), δ 7.44 (C_{α}H-CO, 1H, d, J=15.6 Hz), δ 7.23 (H-6, 1H, dd, J=8.4 Hz and J=1.6 Hz), δ 7.18-7.16 (H-2 and H-5, 2H, m), δ 6.50-6.48 (H-3’and H-5’, 2H, m), δ 5.49 (H-1”, 1H, m), δ 3.97 (H_{α}-5”, 1H, m), δ 3.94 (OCH_3, 3H, s), δ 3.86 (OCH_3, 3H, s), δ 3.63 (H_{β}-5”, 1H, m), δ 2.05-1.57 (H-2”, H3” and H-4”, 6H, m) . ^{13}C NMR (100 MHz, $CDCl_3$): δ 191.81, δ 166.65, δ 166.06, δ 148.45, δ 146.83, δ 144.75, δ 131.11, δ 127.43, δ 123.49, δ 117.77, δ 114.94, δ 114.14, δ 110.20, δ 107.65, δ 101.6, δ 94.68, δ 56.05, δ 55.59, δ 30.70, δ 25.46, δ 19.76. HRMS (ESI- FT-ICR) m/z: [M + H]$^+$ calcd for $C_{22}H_{25}O_6$ 385.16456 (monoisotopic mass), found 385.16469; [M + Na]$^+$ calcd for $C_{22}H_{24}O_6$Na 407.14651 (monoisotopic mass), found 407.14616.

2',4-dihydroxy-3,4'-dimethoxychalcone (**7**) Yellow solid (quantitative yield); mp 135.3-139.2 °C. ^{1}H NMR (400 MHz, $(CD_3)_2CO$) : δ 13.58 (OH, 1H, s), δ 8.18 (OH, 1H, s), δ 8.00 (H-6', 1H, d, J=8.8 Hz), δ 7.73 ($C_{\beta}H$, 1H, d, J=15.2 Hz), δ 7.68 ($C_{\alpha}H$-CO, 1H, d, J=15.2 Hz), δ 7.41 (H-2, 1H, d, J=2 Hz), δ 7.21 (H-6, 1H, dd, J=1.6 Hz and J=8.4 Hz), δ 6.78 (H-5, 1H, d, J=8.4 Hz) , δ 6.38 (H-5', 1H, dd, J=2.4 Hz and J=9.2 Hz), δ 6.33 (H-3', 1H, d, J=2.4 Hz), δ 3.81 (OCH_3, 3H, s), δ 3.76 (OCH_3, 3H, s). ^{13}C NMR (100 MHz, $(CD_3)_2CO$): δ 192.16, δ 166.75, δ 166.26, δ 149.84, δ 147.95, δ145.05, δ 131.84, δ 127.06, δ 124.20, δ 117.47, δ 115.35, δ 114.02, δ 111.22, δ 107.21, δ 100.80, δ 55.54, δ 55.20. HRMS (ESI- FT-ICR) m/z: $[M + H]^+$ calcd for $C_{17}H_{17}O_5$ 301.10705 (monoisotopic mass), found 301.10716; $[M + Na]^+$ calcd for $C_{17}H_{16}O_5Na$ 323.08899 (monoisotopic mass), found 323.08904.

2'-hydroxy-4'-methoxy-4-(tetrahydropyran-2-yloxy)chalcone (**8a**) Yellow solid (quantitative yield); mp 120.8-123.4 °C. ^{1}H NMR (400 MHz, $CDCl_3$): δ 13.54 (OH, 1H, s) 7.87 ($C_{\beta}H$, 1H, d, J=15.6 Hz), δ 7.83 (H-6', 1H, d, J=10.4 Hz), δ 7.61-7.59 (H-2 and H-6, 2H, m), δ 7.47 ($C_{\alpha}H$-CO, 1H, d, J=15.6 Hz), δ 7.11-7.09 (H-3 and H-5, 2H, m), δ 6.50-6.48 (H-3'and H-5', 2H, m), δ 5.50 (H-1", 1H, m), δ 3.89 (H_{α}-5", 1H, m), δ 3.87 (OCH_3, 3H, s), δ 3.63 (H_{β}-5", 1H, m), δ 2.03-1.67 (H-2", H3" and H-4", 6H, m). ^{13}C NMR (100 MHz, $CDCl_3$): δ 191.93, δ 166.64, δ 166.07, δ 159.30, δ 144.32, δ131.14, δ 130.57, δ 130.21, δ 128.24, δ 118.10, δ 116.79, δ 116.02, δ 114.18, δ 107.63, δ 101.09, δ 96.18, δ 62.06, δ 55.58, δ 30.17, δ 25.10, δ 18.57. HRMS (ESI- FT-ICR) m/z: $[M + H]^+$ calcd for $C_{21}H_{23}O_5$ 355.15400 (monoisotopic mass), found 355.15443; $[M + Na]^+$ calcd for $C_{21}H_{22}O_5Na$ 377.13594 (monoisotopic mass), found 377.13542.

2',4-dihydroxy-4'-methoxychalcone (**8**) Yellow solid (yield 70%); mp 152.8-158.5 °C. ^{1}H NMR (400 MHz, $(CD_3)_2CO$): δ 13.72 (OH, 1H, s), δ 9.08 (OH, 1H, s), δ 8.18 (H-6', 1H, d, J=8.8 Hz), δ 7.88 ($C_{\beta}H$, 1H, d, J=15.2 Hz), δ 7.80 ($C_{\alpha}H$-CO, 1H, d, J=15.6 Hz), δ 7.77 (H-2 and H-6, 2H, d, J=8.4 Hz), δ 6.95 (H-3 and H-5, 2H, dd, J=8.4 Hz), δ 6.55 (H-5', 1H, dd, J=2.4 Hz and J=9.2 Hz), δ 6.48 (H-3', 1H, d, J=2.4 Hz), δ 3.90 (OCH_3, 3H, s). ^{13}C NMR (100 MHz, $(CD_3)_2CO$) : δ 192.19, δ 166.72, δ 166.27, δ 160.20, δ 144.59, δ131.87, δ 130.99, δ 126.67, δ 117.36, δ 115.91, δ 114.04, δ 107.17, δ 100.84, δ 55.17. HRMS (ESI- FT-ICR) m/z: $[M + H]^+$ calcd for $C_{16}H_{15}O_4$ 271.09649 (monoisotopic mass), found 271.09629; $[M + Na]^+$ calcd for $C_{16}H_{14}O_4Na$ 293.07843 (monoisotopic mass), found 293.07842.

2',4-dihydroxy-4'-methoxydihydrochalcone (**8b**) Brown oil (yield 10%). ^{1}H NMR (400 MHz, $(CDCl_3)$: δ 12.81 (OH, 1H, s), δ 7.63 (H-6', 1H, d, J=9.6 Hz), δ 7.11 (H-3 and H-5, 2H, d, J=8.4 Hz), δ 6.77 (H-2 and H-6, 2H, d, J=8.4), δ 6.41 (H-3' and H-5', 2 H, m), δ 4.80 (OH, 1H, s), δ 3.83 (OCH_3, 3H, s), δ

3.20 ($C_αH_2$-CO, 2H, t, J=8 Hz), δ 2.98 ($C_βH_2$, 2H, t, J=8 Hz). ^{13}C NMR (100 MHz, ($CDCl_3$)): δ 164.98, δ 164.36, δ 131.98, δ 130.46, δ 128.50, δ114.35, δ 106.66, δ 99.91, δ 54.55, δ 38.92, δ 30.91, δ 28.68, δ 28.51, δ 21.68, δ 13.11, δ 0.00. HRMS (ESI- FT-ICR) m/z: $[M + H]^+$ calcd for $C_{16}H_{17}O_4$ 273.11214 (monoisotopic mass), found 273.11180; $[M + Na]^+$ calcd for $C_{16}H_{16}O_4Na$ 295.09408 (monoisotopic mass), found 295.09452.

2'-hydroxy-4'-methoxychalcone (**9**) Yellow solid (yield 87%); mp 107.5-107.8 °C. ^{1}H NMR (400 MHz, $CDCl_3$): δ 13.43 (OH, 1H, s), δ 7.89 ($C_βH$, 1H, d, J=15.6 Hz), δ 7.84 (H-6', 1H, d, J=8.4 Hz), δ 7.67-7.65 (H-2 and H-6, 2H, m), δ 7.65 ($C_αH$-CO, 1H, d, J=15.6 Hz), δ 7.44-7.42 (H-3, H-4 and H-5, 3H, m), δ 6.51-6.48 (H-3'and H-5', 2H, m), δ 3.87 (OCH_3, 3H, s). ^{13}C NMR (100 MHz, $CDCl_3$) : δ 191.40, δ 166.27, δ 165.80, δ 143.95, δ 134.36, δ 130.78, δ 130.19, δ 128.53, δ 128.07, δ 119.90, δ 113.65, δ 107.31, δ 100.64, δ 55.14. HRMS (ESI- FT-ICR) m/z: $[M + H]^+$ calcd for $C_{16}H_{15}O_3$ 255.10157 (monoisotopic mass), found 255.10180; $[M + Na]^+$ calcd for $C_{16}H_{14}O_3Na$ 277.08352 (monoisotopic mass), found 277.08343.

2'-hydroxy-3,4'-dimethoxychalcone (**10**) Yellow solid (yield 35%); mp 89.2-91.8°C. ^{1}H NMR (400 MHz, $(CD_3)_2CO$): δ 13.56 (OH, 1H, s), δ 8.21 (H-6', 1H, d, J= 8.8 Hz), δ 7.99 ($C_βH$, 1H, d, J=15.2 Hz,), δ 7.88 ($C_αH$, 1H, d, J= 15.6 Hz), δ 7.46-7.38 (H-2, H-4 and H-5, 3H, m), δ 7.05 (H-6, 1H, dd, J=8 Hz and J=2 Hz), δ 6.55 (H-5', 1H, dd, J= 9.2 Hz and J=2.4 Hz), δ 6.50 (H-3', 1H, d, J= 2.4 Hz), δ 3.91 (OCH_3, 3H, s); δ 3.89 (OCH_3, 3H, s). ^{13}C NMR (100 MHz, $(CD_3)_2CO$): δ 192.16, δ 166.84, δ 166.57, δ 160.26, δ 144.24, δ136.36, δ 132.17, δ 129.94, δ 121.58, δ 120.96, δ 116.68, δ 113.97, δ 113.40, δ 107.41, δ 100.83, δ 55.26, δ 54.84. HRMS (ESI- FT-ICR) m/z: $[M + H]^+$ calcd for $C_{17}H_{17}O_4$ 285.11214 (monoisotopic mass), found 285.11230; $[M + Na]^+$ calcd for $C_{17}H_{16}O_4Na$ 307.09408 (monoisotopic mass), found 307.09351.

2'-hydroxy-4'-methoxy-3-(tetrahydropyran-2-yloxy)chalcone (**11a**) Yellow solid (yield 83.44%); mp 92.4-94.6 °C. ^{1}H NMR (400 MHz, $(CD_3)_2CO$): δ 12.77 (OH, 1H, s), δ 7.45 (H-6', 1H, d, J=8.8 Hz) δ 7.20 ($C_βH$, 1H, d, J=15.6 Hz), δ 7.09 ($C_αH$-CO, 1H, d, J=15.6 Hz), δ 6.76 (H-2, 1H, s), δ 6.70 (H-4, 1H, d, J=8 Hz), δ 6.62 (H-5, 1H, t, J=8 Hz), δ 6.4 (H-6, 1H, dd, J=2.4 Hz and J=8.4 Hz), δ 5.78 (H-5', 1H, dd, J=2.4 Hz and J=9.2 Hz), δ 5.72 (H-3', 1H, d, J=2.4 Hz), δ 4.79 (H-1", 1H, t, J=3.2 Hz), δ 3.13 (OCH_3, 3H, s), δ 3.12-3.06 ($H_α$-5", 1H, m), δ 2.86-2.01 ($H_β$-5", 1H, m), δ 1.22-0.82 (H-2", H3" and H-4", 6H, m). ^{13}C NMR (100 MHz, $(CD_3)_2CO$): δ 192.14, δ 166.83, δ 166.57, δ 157.65, δ 144.18, δ 136.24, δ 132.20, δ 129.88, δ 122.39, δ 120.94, δ 118.81, δ 116.38, δ 113.97, δ 107.43, δ 100.82, δ

96.07, δ 61.57, δ 55.26, δ 30.12, δ 25.04, δ 18.64. HRMS (ESI- FT-ICR) m/z: $[M + H]^+$ calcd for $C_{21}H_{23}O_5$ 355.15400 (monoisotopic mass), found 355.15396; $[M + Na]^+$ calcd for $C_{21}H_{22}O_5Na$ 377.13594 (monoisotopic mass), found 377.13506.

2',3-dihydroxy-4'-methoxychalcone (**11**) Yellow solid (yield 10.7 %); mp 174.6-177.8 °C. ^{1}H NMR (400 MHz, $(CD_3)_2CO$): δ 12.71 (OH, 1H, s), δ 7.82 (OH, 1H, s), δ 7.38 (H-6', 1H, d, J=8.8 Hz), δ 7.08 ($C_\beta H$, 1H, d, J=15.6 Hz), δ 7.00 ($C_\alpha H$-CO, 1H, d, J=15.6 Hz), δ 6.49 (H-2, H-4 , H-5; 3H, m, J=8 Hz), δ 6.13 (H-6, 1H, dd, J=2 Hz and J=7.6 Hz) , δ 5.73 (H-5', 1H, dd, J=2.4 Hz and J=9.2 Hz), δ 5.65 (H-3', 1H, d, J=2.4 Hz) δ 3.07 (OCH_3, 3H, s). ^{13}C NMR (100 MHz, $(CD_3)_2CO$): δ 192.18, δ 166.80, δ 166.54, δ 157.88, δ 144.38, δ136.37, δ 132.15, δ 129.98, δ 120.69, δ 120.33, δ 117.87, δ 115.27, δ 113.97, δ 107.39, δ 100.84, δ 55.26. HRMS (ESI- FT-ICR) m/z: $[M + H]^+$ calcd for $C_{16}H_{15}O_4$ 271.09649 (monoisotopic mass), found 271.09660; $[M + Na]^+$ calcd for $C_{16}H_{14}O_4Na$ 293.07843 (monoisotopic mass), found 293.07868.

2,2'-dihydroxy-4'-methoxychalcone (**12**) Yellow solid (yield 4%); mp 159.9-164.0 °C. ^{1}H NMR (400 MHz, $(CD_3)_2CO$): δ 13.70 (OH, 1H, s), δ 9.30 (OH, 1H, s), δ 8.33 ($C_\beta H$, 1H, d, J=15.6 Hz), δ 8.17 (H-6', 1H, d, J=9.2 Hz), δ 8.03 ($C_\alpha H$-CO, 1H, d, J=15.6 Hz), δ 7.89 (H-6, 1H, dd, J=1.6 Hz and J=8.4 Hz), δ 7.36-7.32 (H-4, 1H, m), δ 7.05 (H-3, 1H, dd, J=2.8 Hz and J=8 Hz) , δ 6.98 (H-5, 1H, t, J=7.6 Hz), δ 6.59 (H-5', 1H, dd, J=2.4 Hz and J=8.8 Hz,), δ 6.52 (H-3', 1H, d, J=2.4 Hz), δ 3.93 (OCH_3, 3H, s). ^{13}C NMR (100 MHz, $(CD_3)_2CO$): δ 193.73, δ 192.54, δ 166.77, δ 166.35, δ 157.20, δ 148.05, δ139.83, δ 131.96, δ 129.16, δ 121.94, δ 120.03, δ 116.27, δ 114.07, δ 107.29, δ 100.86, δ 55.22. HRMS (ESI- FT-ICR) m/z: $[M + H]^+$ calcd for $C_{16}H_{15}O_4$ 271.09649 (monoisotopic mass), found 271.09631; $[M + Na]^+$ calcd for $C_{16}H_{14}O_4Na$ 293.07843 (monoisotopic mass), found 293.07863.

2'-hydroxy-2,4'-dimethoxychalcone (**13**) Yellow solid (yield 46%); mp 74.7-77.9°C. ^{1}H NMR (400 MHz, $(CD_3)_2CO$): δ 12.87 (OH, 1H, s), δ 7.51 ($C_\beta H$, 1H, d, J=15.6 Hz), δ 7.40 (H-6', 1H, d, J=8.8 Hz), δ 7.21 ($C_\alpha H$-CO, 1H, d, J=15.6 Hz), δ 7.16 (H-6, 1H, dd, J=7.6 Hz and J=0.8 Hz), δ 6.70 (H-4, 1H, td, J=1.2 Hz and J= 8.0 Hz), δ 6.37 (H-3, 1H, d, J=8.4 Hz), δ 6.28 (H-5, 1H, d, J=7.6 Hz), δ 5.78 (H-5', 1H, dd, J=2.4 Hz and J=9.2 Hz), δ 5.72 (H-3', 1H, d, J=2.4 Hz), δ 3.22 (OCH_3, 3H, s), δ 3.14 (OCH_3, 3H, s). ^{13}C NMR (100 MHz, $(CD_3)_2CO$): δ 192.44, δ 166.80, δ 166.42, δ 158.93, δ 139.26, δ 132.30, δ 131.99, δ 128.88, δ 123.48, δ 120.72, δ 120.59, δ 114.04, δ 111.56, δ 107.34, δ 100.85, δ 55.24, δ 54.64. HRMS (ESI- FT-ICR) m/z: $[M + H]^+$ calcd for $C_{17}H_{17}O_4$ 285.11214 (monoisotopic mass), found 285.11237; $[M + Na]^+$ calcd for $C_{17}H_{16}O_4Na$ 307.09408 (monoisotopic mass), found 307.09351.

2’-hydroxy-4-(tetrahydropyran-2-yloxy)chalcone (**14a**) Yellow solid (yield 64%); mp 138.6-139.0°C. ^{1}H NMR (400 MHz, $(CD_3)_2CO$): δ 12.24 (OH, 1H, s), δ 7.47 (H-6’, 1H, d, J=7.6 Hz), δ 7.16 ($C_{\beta}H$, 1H, d, J=15.6 Hz), δ 7.12 ($C_{\alpha}H$-CO, 1H, d, J=15.6 Hz) δ 7.06 (H-2 and H-6, 2H, d, J=8.8 Hz), δ 6.76 (H-4’, 1H, t, J=8 Hz), δ 6.34 (H-3 and H-5, 2H, d, J=8.4 Hz), δ 6.21-6.17 (H-3’and H-5’, 2H, m), δ 4.77 (H-1”, 1H, t, J=3.2 Hz), δ 3.05-2.99 (H_{α}-5”, 1H, m), δ 32.83-2.74 (H_{β}-5”, 1H, m), δ 1.16-0.78 (H-2”, H3” and H-4”, 6H, m) . ^{13}C NMR (100MHz, $(CD_3)_2CO$) : δ 194.04, δ 163.64, δ 159.70, δ 145.34, δ 136.34, δ 130.80, δ 130.35, δ 128.14, δ 120.05, δ 118.86, δ 118.17, δ 116.74, δ 107.43, δ 95.97, δ 61.63, δ 56.84, δ 29.97, δ 24.95, δ 18.51, δ 18.01. HRMS (ESI- FT-ICR) m/z: $[M + H]^+$ calcd for $C_{20}H_{21}O_4$ 325.14344 (monoisotopic mass), found 325.14319; $[M + Na]^+$ calcd for $C_{20}H_{20}O_4Na$ 347.12538 (monoisotopic mass), found 347.12539.

2’,4-dihydroxychalcone (**14**) Yellow solid (yield 60%); mp 148.8-150.2°C. ^{1}H NMR (400 MHz, $(CD_3)_2CO$) : δ 12.15 (OH, 1H, s), δ 8.17 (OH, 1H, s), δ 7.31 (H-6’, 1H, d, J=7.2 Hz) δ 6.98 ($C_{\beta}H$, 1H, d, J=15.6 Hz), δ 6.93 ($C_{\alpha}H$-CO, 1H, d, J=15.2 Hz), δ 6.85 (H-2 and H-6, 2H, d, J=8.8 Hz), δ 6.60 (H-4’, 1H, t, J=7.2 Hz), δ 6.05-5.99 (H-3, H-3’, H-5 and H-5’, 4H, m). ^{13}C NMR (100 MHz, $(CD_3)_2CO$): δ 194.02, δ 163.62, δ 160.51, δ 145.79, δ 136.21, δ 131.28, δ 130.26, δ 126.50, δ 120.09, δ 118.81, δ 118.03, δ 117.10, δ 115.99. HRMS (ESI- FT-ICR) m/z: $[M + H]^+$ calcd for $C_{15}H_{13}O_3$ 241.08592 (monoisotopic mass), found 241.08620; $[M + Na]^+$ calcd for $C_{15}H_{12}O_3Na$ 263.06787 (monoisotopic mass), found 263.06769.

2’-methoxy-4-(tetrahydropyran-2-yloxy)chalcone (**15a**) Yellow solid (yield 38%); mp 83.7-89.3°C. ^{1}H NMR (400 MHz, $(CD_3)_2CO$): δ 6.88 (H-2 and H-6, 2H, d, J=8.4 Hz), δ 6.78-6.72 ($C_{\beta}H$, H-4’ and H-6’, 3H, m), δ 6.57 ($C_{\alpha}H$-CO, 1H, d, J=16 Hz), δ 6.38 (H-3’, 1H, d, J=8 Hz), δ 6.32 (H-3 and H-5, 2H, d, J=8.8 Hz), δ 6.27 (H-5’, 1H, d, J=7.2 Hz), δ 4.75 (H-1”, 1H, t, J=3.2 Hz), δ 3.14 (OCH_3, 3H, s), δ 3.06-3.01 (H_{α}-5”, 1H, m), δ 2.83-2.78 (H_{β}-5”, 1H, m), δ 1.28-0.79 (H-2”, H3” and H-4”, 6H, m). ^{13}C NMR (100 MHz, $(CD_3)_2CO$): δ 191.69, δ 159.05, δ 158.15, δ 142.08, δ 132.63, δ 129.91, δ 129.81, δ 129.75, δ 128.52, δ 125.24, δ 120.52, δ 116.72, δ 111.94, δ 96.01, δ 61.63, δ 55.28, δ 30.02, δ 24.98, δ 18.57. HRMS (ESI- FT-ICR) m/z: $[M + H]^+$ calcd for $C_{21}H_{23}O_4$ 339.15909 (monoisotopic mass), found 339.15784; $[M + Na]^+$ calcd for $C_{21}H_{22}O_4Na$ 361.14103 (monoisotopic mass), found 361.14116.

4-hydroxy-2’-methoxychalcone (**15**) Yellow solid (yield 82%); mp 135.3-138.0 °C. ^{1}H NMR (400 MHz, $(CD_3)_2CO$): δ 8.05 (OH, 1H, s), δ 6.66 (H-2 and H-6, 2H, d, J=8.8 Hz), δ 6.61-6.57 ($C_{\beta}H$, H-4’ and H-6’, 3H, m), δ 6.36 ($C_{\alpha}H$-CO, 1H, d, J=16 Hz), δ 6.23 (H-3’, 1H, d, J=8 Hz), δ 6.12 (H-5’, 1H, t,

J=7.6 Hz), δ 5.99 (H-3 and H-5, 2H, d, J=8.4 Hz), δ 2.99 (OCH_3, 3H, s).^{13}C NMR (100 MHz, $(CD_3)_2CO$): δ 191.81, δ 159.78, δ 158.07, δ 142.66, δ 132.48, δ 130.34, δ 129.88, δ 129.74, δ 126.77, δ 124.30, δ 120.49, δ 115.90, δ 111.92, δ 55.26. HRMS (ESI- FT-ICR) m/z: $[M + H]^+$ calcd for $C_{16}H_{15}O_3$ 255.10157 (monoisotopic mass), found 255.10180; $[M + Na]^+$ calcd for $C_{16}H_{14}O_3Na$ 277.08352 (monoisotopic mass), found 277.08348.

2',4'-dimethoxy-4-(tetrahydropyran-2-yloxy)chalcone (**16a**) Yellow solid (yield 15%); mp 134.7-135.9 °C. ^1H NMR (400 MHz, ($CDCl_3$)): δ 7.73 (H-6', 1H, d, J=8 Hz), δ 7.64 ($C_\beta H$, 1H, d, J=16 Hz), δ 7.53 (H-2 and H-6, 2H, d, J=8 Hz), δ 7.38 ($C_\alpha H$-CO, 1H, d, J=16 Hz), δ 7.05 (H-3 and H-5, 2H, d, J= 8.8 Hz), δ 6.56 (H-5', 1H, dd, J=2.4 Hz, J=8 Hz), δ 6.49 (H-3', 1H, d, J=2.4 Hz), δ 5.47 (H-1", 1H, t, J=2.8 Hz), δ 3.90-3.87 (H_α-5", OCH_3, 7H, m), δ 3.61 (H_β-5", 1H, m), δ 2.03-1.59 (H-2", H-3" and H-4", 6H, m). ^{13}C NMR (100 MHz, $CDCl_3$): δ 190.75, δ 163.98, δ 160.28, δ 158.71, δ142.14, δ 132.76, δ 129.87, δ 128.90, δ 125.26, δ 122.49, δ 116.65, δ 105.10, δ 98.70, δ 96.18, δ 62.07, δ 55.77, δ 55.55, δ 30.22, δ 25.13, δ 18.64. HRMS (ESI- FT-ICR) m/z: $[M + H]^+$ calcd for $C_{22}H_{25}O_5$ 369.16965 (monoisotopic mass), found 369.16998; $[M + Na]^+$ calcd for $C_{22}H_{24}O_5Na$ 391.15159 (monoisotopic mass), found 391.15055.

4-hydroxy-2',4'-dimethoxychalcone (**16**) Yellow solid (yield 92%); mp 139.9-142.2 °C. ^1H NMR (400 MHz, ($CDCl_3$)) : δ 12.87 (OH, 1H, s), δ 7.51 ($C_\beta H$, 1H, d, J=15.6 Hz), δ 7.40 (H-6', 1H, d, J=8.8 Hz), δ 7.21 ($C_\alpha H$-CO, 1H, d, J=15.6 Hz), δ 7.16 (H-6, 1H, dd, J=7.6 Hz, J=0.8 Hz), δ 6.70 (H-4, 1H, td, J= 8.0 Hz, J=1.2 Hz), δ 6.37 (H-3, 1H, d, J=8.4 Hz) , δ 6.28 (H-5, 1H, d, J=7.6 Hz), δ 5.78 (H-5', 1H, dd, J=9.2 Hz, J=2.4 Hz), δ 5.72 (H-3', 1H, d, J=2.4 Hz), δ 3.22 (OCH_3, 3H, s), δ 3.14 (OCH_3, 3H, s). ^{13}C NMR (100 MHz, $CDCl_3$): δ 191.31, δ 164.13, δ 160.38, δ 158.10, δ 142.81, δ 132.80, δ 130.32, δ 127.89, δ 124.68, δ 122.29, δ 115.98, δ 105.14, δ 98.75, δ 55.77, δ 55.57. HRMS (ESI- FT-ICR) m/z: $[M + H]^+$ calcd for $C_{17}H_{17}O_4$ 285.11214 (monoisotopic mass), found 285.11192; $[M + Na]^+$ calcd for $C_{17}H_{16}O_4Na$ 307.09408 (monoisotopic mass), found 307.09369.

4'-methoxy-4-(tetrahydropyran-2-yloxy)chalcone (**17a**) Yellow solid (yield 66%); mp 134.7-135.9 °C. ^1H NMR (400 MHz, ($CDCl_3$)): δ 8.03 (H-2 and H-6, 2H, d, J=8.8 Hz), δ 7.78 ($C_\beta H$, 1H, d, J=15.6 Hz), δ 7.58 (H-2' and H-6', 2H, d, J=8.8 Hz), δ 7.43 ($C_\alpha H$-CO, 1H, d, J=15.6 Hz), δ 7.08 (H-3 and H-5, 2H, d, J= 8.4 Hz), δ 6.99 (H-3' and H-5', 2H, d, J= 8.4 Hz), δ 5.48 (H-1", 1H, t, J=2.8 Hz), δ 3.91-3.85 (H_α-5", 1H, m), δ 3.89 (OCH_3, 3H, s), δ 3.65-3.60 (H_β-5", 1H, m), δ 2.02-1.60 (H-2", H-3" and H-4", 6H, m). ^{13}C NMR (100 MHz, $CDCl_3$): δ 188.84, δ 163.29, δ 159.00, δ 143.88, δ 131.37, δ 130.73, δ 129.98

δ 128.53, δ 119.81, δ 116.72, δ 113.80, δ 96.17, δ 62.06, δ 55.50, δ 30.20, δ 25.13, δ 18.61. HRMS (ESI- FT-ICR) m/z: $[M + H]^+$ calcd for $C_{21}H_{23}O_4$ 339.15909 (monoisotopic mass), found 339.15756; $[M + Na]^+$ calcd for $C_{21}H_{22}O_4Na$ 361.14103 (monoisotopic mass), found 361.14116.

4-hydroxy-4'-methoxychalcone (**17**) Yellow solid (yield 96%); mp 171.3-175.8 °C. ^1H NMR (400 MHz, $(CD_3)_2CO$): δ 8.17 (H-2 and H-6, 2H, d, J=8.8 Hz), δ 7.77 ($C_\beta H$, 1H, d, J=15.6 Hz), δ 7.79-7.71 (H-2'and H-6', 2H, m), δ 7.73 ($C_\alpha H$-CO, 1H, d, J=16 Hz), δ 7.10 (H-3' and H-5', 2H, d, J=8.8 Hz), δ 6.96 (H-3 and H-5, 2H, d, J=8.8 Hz), δ 3.94 (OCH_3, 3H, s). ^{13}C NMR (100 MHz, $(CD_3)_2CO$): δ 187.24, δ 163.39, δ 159.86, δ 143.40, δ 131.43, δ 130.55, δ 130.51, δ 126.88, δ 118.71, δ 115.86, δ 113.75, δ 55.03. HRMS (ESI- FT-ICR) m/z: $[M + H]^+$ calcd for $C_{16}H_{15}O_3$ 255.10157 (monoisotopic mass), found 255.10177; $[M + Na]^+$ calcd for $C_{16}H_{14}O_3Na$ 277.08352 (monoisotopic mass), found 277.08332.

Chromatographic conditions for the purity check

HPLC analytical separations were performed on a Waters 2690 Separation Module, equipped with a Rheodyne Model 8125 20-μl injector and a Model M486 programmable multi-wavelength detector (PDA). Chromatographic data were collected and processed using the Empower Chromatography Manager software. Purity of the sample used in this study was higher than 95% by HPLC.

Compound **A**, Column: Phenomenex Luna C18, 5.0 μm (150 × 4.6 mm). Eluent A) water solution of trifluoroacetic acid 0.03% (v/v). Eluent B) acetonitrile. Gradient elution: 0-1 min 80%A-20%B; 1-10 min 60%A-40%B; 10-20 min 100%B; 20-25 min 80%A-20%B. Flow rate: 1.0 ml/min. PDA detection at 290 nm. Retention time (R_t) = 7.90 min.

Compound **B**, Column: Phenomenex Luna C18, 5.0 μm (250 × 4.6 mm). Eluent A) water/acetonitrile = 95:5 (v/v). Eluent B) water/acetonitrile = 5:95 (v/v). Gradient elution: for 0-5 min A:B = 50:50; 5-20 min up to 100% B; 20-25 min to 100% B. Flow rate: 1.0 ml/min. PDA detection at 295 nm. Retention time (R_t) = 13.12 min.

Compounds **E**, Column: Phenomenex Luna C18, 5.0 μm (250 × 4.6 mm). Eluent A) methanol. Eluent B) water. Gradient elution: 0–6 min A:B = 65:35; 6–8 min up to 81% A; 8–15 min A:B = 81:19; 15-20 min up to 65% A. PDA detection at 325 nm. Retention time (R_t) = 5.02 min.

Compound **F**, Column: Phenomenex Luna C18, 5 μm (250 × 4.6 mm). Eluent: methanol/ water = 90:10 (v/v). Flow rate: 1.0 ml/min. PDA detection at 237 nm. Retention time (R_t) = 5.83 min.

For chalcone derivatives **1 – 17**, HPLC conditions, % and Retention times (R_t) were as follows. Column: Phenomenex Luna C18, 5.0 μm (250 × 4.6 mm). Eluent A) water/acetonitrile = 95:5 (v/v). Eluent B) water/acetonitrile = 5:95 (v/v). Gradient elution: for 0-5 min A:B = 50:50; 5-20 min up to 100% B; 20-25 min to 100% B. Flow rate: 1.0 ml/min. PDA detection at 200-400 nm.For compound **1**, 95.01% R_t = 11.88 min (at 295 nm); for compound **2**, 98.41% R_t = 15.67 min (at 295 nm); for compound **3**, 98.92 R_t = 17.98 min (at 295 nm); for compound **4**, 99.29% R_t = 14.76 min (at 295 nm); for compound **5a**, 98.28% R_t = 19.44 min (at 295 nm); for compound **5**, 99.76% R_t = 13.39 min (at 295 nm); for compound **6a**, 100% R_t = 19.45 min (at 295 nm); for compound **7a**, 99.85 % R_t = 19.26 min (at 295 nm); for compound **7**, 99.36% R_t = 12.98 min (at 295 nm); for compound **8a**, 99.98% R_t = 20.95 min (at 295 nm); for compound **8**, 98.87% R_t = 12.61 min (at 295 nm); for compound **8b**, 97.40% R_t = 11.94 min (at 295 nm); for compound **9**, 100% R_t = 18.30 min (at 295 nm); for compound **10**, 99.29% R_t = 18.12 min (at 295 nm); for compound **11a**, 100.00% R_t = 21.01 min (at 295 nm); for compound **11**, 96.25% R_t = 13.15 min (at 295 nm); for compound **12**, 95.24% R_t = 13.15 min (at 295 nm); for compound **13**, 99.41% R_t = 18.78 min (at 295 nm); for compound **14a**, 98.68% R_t = 21.01 min (at 295 nm); for compound **14**, 99.31% R_t = 12.77 min (at 295 nm); for compound **15a**, 98.84% R_t = 18.43 min (at 295 nm); for compound **15**, 95.57% R_t = 8.67 min (at 295 nm); for compound **16a**, 100.00% R_t = 17.83 min (at 295 nm); for compound **16**, 99.53% R_t = 8.18 min (at 295 nm); for compound **17a**, 95.00 % R_t = 17.41 min (at 295 nm); for compound **17**, 98.40% R_t = 8.93 min (at 295 nm).

Cell proliferation assay and IC_{50} determination

Cells were seeded in 96 well plate at $5X10^5$ cells/ml ($5X10^4$ cells/well) and then treated at the indicated compounds concentrations and periods. Three technical replicates were used for all treatments including the only culture medium and DMSO treatments. All reactions in the inhibitor experiments contain a final concentration of 1% DMSO. To quantifies the viability, cells were assayed using the MTS-based assay CellTiter 96® AQueous One Solution Cell Proliferation Assay (G3580; Promega, Madison, WI, USA). The absorbance was measured at 490nm by using GloMax Multidetction System (Promega, Madison, WI, USA). Data was collected in the form of units of absorbance (ABS) and normalized to percent cell proliferation (MTS) with the use of equation: % Cell Proliferation = $(ABS_{cells+compound} - ABS_{medium+compound}) / (ABS_{cells+DMSO} - ABS_{medium+ DMSO})$ X 100. The linear portion of

the reaction progress curve thus created was measured to yield an initial rate (Co); a plot of inhibitor concentration (log[compound]) versus % Cell Proliferation was fit by non-linear regression curve analysis to calculate the absolute IC_{50} by using GraphPad Prism software.

Notch reporter assays

Transient transfections were performed by Lipofectamine® 2000 Transfection Reagent (11668027, Invitrogen, Carlsbad, CA, USA) according to the manufacturer's instructions. HEK cells were transfected with Notch-responsive firefly 12X CSL-Luciferase reporter[1] and with pRL-CMV Vector (E2261, Promega, Madison, WI, USA) in combination with pCS2 Notch1 ΔEMV-6MT (N1ICD-ΔE) vector encoding the murine Notch1 leader peptide (aa 1-23) followed by aa 1704-2184 and a hexameric Myc Tag at the C-terminal. pCS2 Notch1 ΔEMV-6MT was a gift from Raphael Kopan & Jeffrey Nye (Addgene plasmid # 41737) and it has been described in [2]. After 12 hrs of transfection cells were further treated for 36 hrs with different doses of DAPT, GI254023X or compound **8.** Luciferase and renilla activity were assayed with a dual-luciferase assay system (E1980; Promega, Madison, WI, USA).

Cell cycle analysis

After each treatment, cells were washed in PBS buffer and suspended in 1 ml of Fixation/Permeabilization solution (555028, BD-Biosciences, San Jose, CA, USA). After 4hrs of incubation, each sample was washed and followed by incubation with 50 μg/ml RNase (R4875, Sigma-Aldrich, St Louis, MO, USA) and 25 μg/ml 7AAD (A 9400, Sigma-Aldrich, St Louis, MO, USA) at room temperature for 12 hours. Cells were then acquired using a flow cytometer. Fluorescence-activated cell sorting (FACS) analysis was performed using BD LSRFortessa equipped with 488 nm laser and with DIVA software (BD Biosciences, San Jose, CA, USA). The cells were first gated using forward vs side scatter (FSC vs SSC) strategy and upon 488nm laser excitation, 7-AAD fluorescence was then detected in the far red range of the spectrum (685nm long pass). Data were analyzed using FlowJo software (FlowJo LLC data analysis software, Ashland, OR, USA).

Apoptosis evaluation by Annexin-V and 7-AAD assay

Apoptosis was detected by using FITC Annexin V Apoptosis Detection Kit with 7-AAD (640922, BioLegend, San Diego, CA, USA). Briefly, cells were washed twice with cold BioLegend's Cell Staining Buffer, suspended in Annexin V Binding Buffer and, then additioned with 5µl of FITC Annexin V and 5µl of 7-AAD Viability Staining Solution. After 15 min of incubation at room temperature in the dark, samples were analyzed using BD LSRFortessa equipped with 488 nm laser and with DIVA software (BD Biosciences, San Jose, CA, USA). The cells were first gated using forward vs side scatter (FSC vs SSC) strategy and upon 488nm laser excitation, 7-AAD fluorescence was then detected in the far red range of the spectrum (685nm long pass) and Annexin V was detected at 530nm emission (FITC channel). Data were analyzed using FlowJo software (FlowJo LLC data analysis software, Ashland, OR, USA).

Retroviral transduction and rescue assay.

Retroviral construct encoding the entire murine N1ICD fragment GFP CMMP-ICN1-IRES-EGFP (mICN1) and the empty relative control vector CMMP-IRES-EGFP (empty) have been generated as previously described [3]. Viral supernatants were produced by transient transfection of retroviral vectors and packaging plasmids [4] into HEK 293T by TransFectin Lipid Reagent (170-3351, Biorad, Hercules, CA, USA), according to manufacturer's instructions. 48 hrs after transfection, conditioned medium containing retroviral particles was collected, filtered through a 0.2 µm membrane, and immediately used for transduction assay. 2×10^6 DND41 cells were centrifuged with the appropriate amount of viral supernatants and 5 µg/mL hexadimethidrine bromide (H9268, Sigma-Aldrich, St Louis, MO, USA) for 90 min at 2200 rpm at room temperature and then washed and plated at a concentration of 5×10^5 cells per ml. 5 days after infection cells were collected, and GFP-transduced cells were FACS sorted to 99% purity. Growth sensitivity to treatment with 1 µM of compound **8** was compared between DND41 transduced with mICN1and with empty retroviruses for times indicated in figure. To assess cell growth, trypan blue (T8154, Sigma-Aldrich, St Louis, MO, USA) was used to count viable cells.

Fluorescence-Activated Cell Sorting

DND4-1 transduced cells were sorted based on GFP expression using a FACSAriaIII (BD Biosciences) equipped with a 488nm laser and FACSDiva software (BD Biosciences version 6.1.3). Briefly, cells

first gated based on forward and side scatter area (FSC-A and SSC-A) plot were then detected in the green fluorescence channel for GFP expression and isolated based on high GFP levels. Upon sorting an aliquot of the collected cells was checked for purity (>99%).

SUPPLEMENTARY REFERENCES

1 Hansson, E. M. *et al.* Recording Notch signaling in real time. *Developmental neuroscience* **28**, 118-127, doi:10.1159/000090758 (2006).

2 Kopan, R., Schroeter, E. H., Weintraub, H. & Nye, J. S. Signal transduction by activated mNotch: importance of proteolytic processing and its regulation by the extracellular domain. *Proceedings of the National Academy of Sciences of the United States of America* **93**, 1683-1688 (1996).

3 Campese, A. F. *et al.* Notch1-dependent lymphomagenesis is assisted by but does not essentially require pre-TCR signaling. *Blood* **108**, 305-310, doi:10.1182/blood-2006-01-0143 (2006).

4 Ory, D. S., Neugeboren, B. A. & Mulligan, R. C. A stable human-derived packaging cell line for production of high titer retrovirus/vesicular stomatitis virus G pseudotypes. *Proceedings of the National Academy of Sciences of the United States of America* **93**, 11400-11406 (1996).
